# Loss of malic enzymes leads to metabolic imbalance and altered levels of trehalose and putrescine in the bacterium *Sinorhizobium meliloti*

**DOI:** 10.1186/s12866-016-0780-x

**Published:** 2016-07-26

**Authors:** Ye Zhang, Laura Anne Smallbone, George C. diCenzo, Richard Morton, Turlough M. Finan

**Affiliations:** 1 Department of Biology, McMaster University, 1280 Main St. West, Hamilton, ON L8S 4K1 Canada; 2 College of Fisheries and Life Science, Shanghai Ocean University, Shanghai, China

**Keywords:** Putrescine, Trehalose, Amino acids, Fatty acids, Malic enzyme, *Sinorhizobium*, Catabolite repression

## Abstract

**Background:**

Malic enzymes decarboxylate the tricarboxylic acid (TCA) cycle intermediate malate to the glycolytic end-product pyruvate and are well positioned to regulate metabolic flux in central carbon metabolism. Despite the wide distribution of these enzymes, their biological roles are unclear in part because the reaction catalyzed by these enzymes can be by-passed by other pathways. The N_2_-fixing alfalfa symbiont *Sinorhizobium meliloti* contains both a NAD(P)-malic enzyme (DME) and a separate NADP-malic enzyme (TME) and to help understand the role of these enzymes, we investigated growth, metabolomic, and transcriptional consequences resulting from loss of these enzymes in free-living cells.

**Results:**

Loss of DME, TME, or both enzymes had no effect on growth with the glycolytic substrate, glucose. In contrast, the *dme* mutants, but not *tme*, grew slowly on the gluconeogenic substrate succinate and this slow growth was further reduced upon the addition of glucose. The *dme* mutant strains incubated with succinate accumulated trehalose and hexose sugar phosphates, secreted malate, and relative to wild-type, these cells had moderately increased transcription of genes involved in gluconeogenesis and pathways that divert metabolites away from the TCA cycle. While *tme* mutant cells grew at the same rate as wild-type on succinate, they accumulated the compatible solute putrescine.

**Conclusions:**

NAD(P)-malic enzyme (DME) of *S. meliloti* is required for efficient metabolism of succinate via the TCA cycle. In *dme* mutants utilizing succinate, malate accumulates and is excreted and these cells appear to increase metabolite flow via gluconeogenesis with a resulting increase in the levels of hexose-6-phosphates and trehalose. For cells utilizing succinate, TME activity alone appeared to be insufficient to produce the levels of pyruvate required for efficient TCA cycle metabolism. Putrescine was found to accumulate in *tme* cells growing with succinate, and whether this is related to altered levels of NADPH requires further investigation.

**Electronic supplementary material:**

The online version of this article (doi:10.1186/s12866-016-0780-x) contains supplementary material, which is available to authorized users.

## Background

Glucose is catabolized to pyruvic acid via the Embden-Meyerhof-Parnas (EMP) and/or the Entner-Doudoroff (ED) glycolytic pathways in many microorganisms. The pentose phosphate pathway is also used in both catabolic and anabolic metabolism. In aerobic organisms, pyruvate is oxidized via the tricarboxylic acid (TCA) cycle and the resulting reductant is passed-on to the electron acceptor O_2_ through an electron transport chain (ETC). This process generates an electrochemical proton potential that is used by the proton ATPase for ATP synthesis. When TCA cycle intermediates such as succinate or malate are employed as the carbon source, energy is generated via the TCA cycle and the ETC, while glucose and other sugars required for biosynthetic reactions are synthesized by a reversal of many of the reactions of the glycolytic pathways. This process is referred to as gluconeogenesis. Because several glycolytic reactions are not reversible, several new enzymes, such as phosphoenoylpyruvate (PEP) carboxykinase are synthesized in order for gluconeogenesis to occur. In both glycolysis and gluconeogenesis, the metabolic intermediates acetyl-CoA, pyruvate, PEP, 2-ketoglutarate, succinyl-CoA, and oxaloacetate are precursors for the synthesis of amino acids, nucleotides, lipids, and tetrapyrroles. The removal of these precursor compounds reduces flux through those pathways, and for flux through central carbon pathways to be maintained the lost intermediates are replenished via anaplerotic “fill-in” reactions catalyzed by enzymes such as pyruvate carboxylase, phosphoenolpyruvate carboxylase, and malic enzymes [1, 2].

Malic enzymes (ME) decarboxylate L-malate to pyruvate and reduce NAD(P)^+^ to NAD(P)H + H^+^ [3–5]. They link pathways of glycolysis and gluconeogenesis with the TCA cycle and play an anapleorotic role in replenishing metabolic intermediates and in the generation of NAD(P)H reductant [6]. There is considerable variation in the distribution and the properties of MEs found in bacteria; some carry both NAD and NADP-dependent enzymes while other carry a single ME enzyme [3, 5, 7–9].

Rhizobia are free-living soil bacteria that form N_2_-fixing nodules on the roots of leguminous plants. The alfalfa symbiont *Sinorhizobium meliloti* has two MEs. DME is a diphosphopyridine nucleotide (NAD^+^)-dependent ME that also has some activity with NADP^+^ (EC 1.1.1.39), whereas TME is a strictly triphosphopyridine nucleotide (NADP^+^)-dependent ME, (EC 1.1.1.40) [3, 10–12]. The *S. meliloti* DME and TME proteins share similar kinetic properties (*K*_*m*_, *V*_*max*_) for L-malate and their respective co-factors NAD^+^ or NADP^+^ respectively. DME activity is allosterically activated by malate, succinate, and fumarate, and is inhibited by acetyl-CoA, whereas TME activity does not appear to be regulated [11]. DME, TME, and the *Escherichia coli* NADP^+^-dependent ME (*maeB*) are large 700 amino acids proteins. Their 400-amino-acid long N-terminal regions are responsible for the ME activity. The 300-amino-acid C- terminal domain is similar in sequence to phosphotransacetylase enzymes (PTA) but no PTA activity has been detected and the function of this large domain is unclear [3, 5].

The ME domain of the *S. meliloti* DME protein is required for N_2_-fixation in alfalfa nodules [13] and this requirement cannot be replaced through the production of the TME enzyme [13]. The C_4_-dicarboxylates succinate and malate appear to be the primary carbon and energy sources used by the N_2_-fixing bacteria in the nodules [14–16], and it is thought that DME is required to synthesize high levels of pyruvate for use by pyruvate dehydrogenase (PDH) in the generation of acetyl-CoA to generate the energy necessary for the ATP intensive nitrogenase reaction [12, 16]. In some symbioses, pyruvate and acetyl-CoA can be produced through an alternate route catalyzed by the enzymes PEP-carboxykinase, pyruvate kinase, and PDH [17–19].

Here, we further investigated the biological roles of the DME and TME malic enzymes by investigating the transcription and polar metabolite profiles, and growth phenotypes of *S. meliloti* free-living *dme* and/or *tme* mutant cells. The findings are discussed with respect to the role of malic enzymes in central carbon metabolism and the role of DME in N_2_-fixing nodules.

## Results

### Global metabolite analysis

**Fig. 1:**
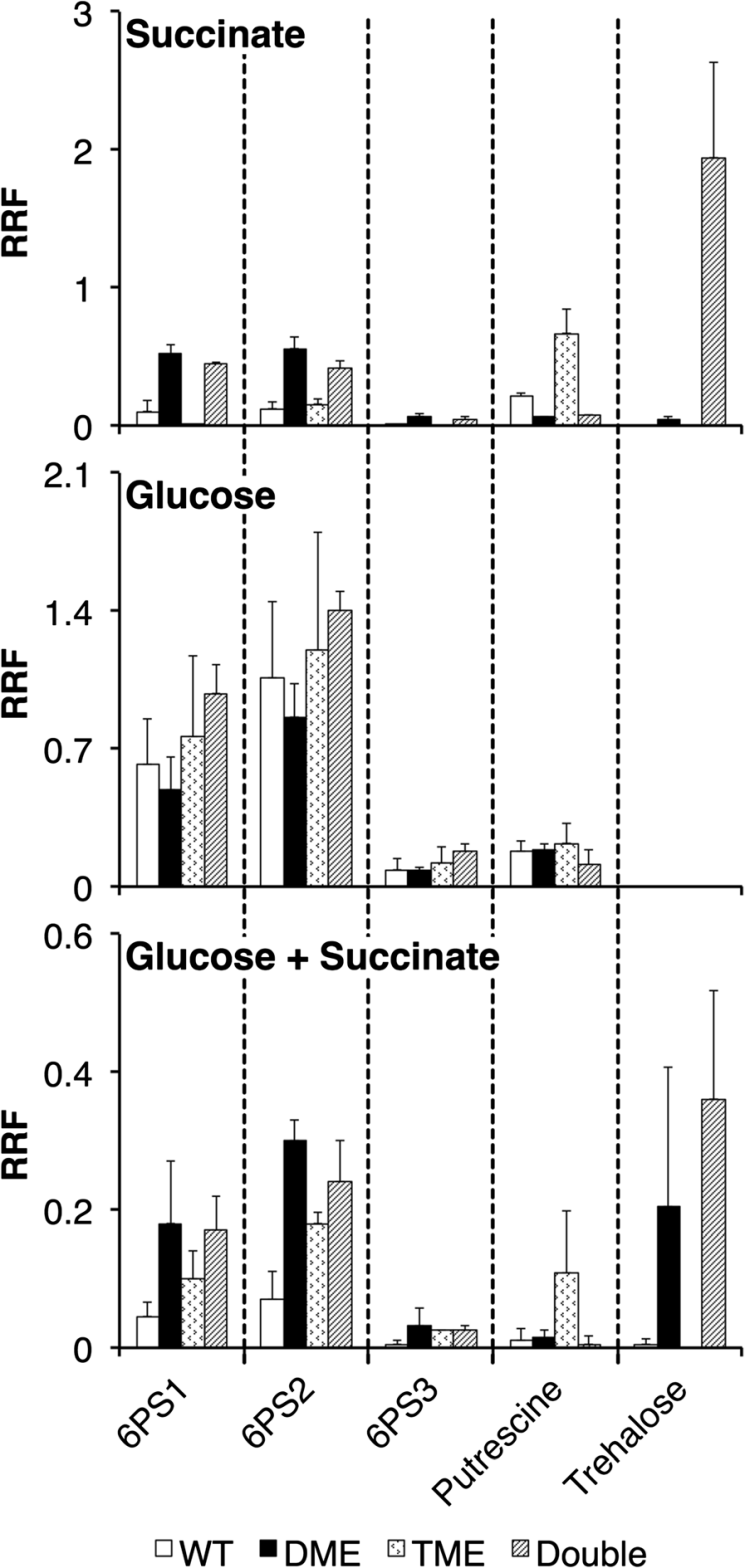
Relative response factors (RRF) for intracellular metabolites with significantly different RRFs (*P* values of < 0.05 in ANOVA) from the wild-type strain, *dme*, *tme* and *dme tme* double mutants. Note the different RRF scale for the three growth conditions. Metabolites were 6-phospho-sugars (likely fructose-6-phosphate (6PS1), mannose-6-phosphate (6PS2) and glucose-6-phosphate (6PS3)), putrescine and trehalose. Strains were grown in M9-Succinate, M9-Glucose, M9-Succinate plus Glucose. The trehalose RRFs were negligible in extracts for strains grown in M9-glucose. For M9-Succinate + Glucose, the cells grown in Succinate + Glucose were washed and incubated for 2 h in modified M9-Succinate. Error bars were calculated using standard deviation of the mean from three independent cultures

To identify metabolic differences that may result from malic enzyme mutations, intracellular polar metabolites from cultures grown with either a glycolytic (glucose) or gluconeogenic (succinate) carbon source were analyzed by GC-MS. Metabolites were analyzed from wild type and *dme* or *tme* mutant strains. In addition a *dme tme* double mutant was examined to investigate whether the removal of both malic enzymes exaggerates the metabolic defects. No metabolite differences were detected when glucose-grown cells of the wild-type were compared with glucose-grown *dme* or *tme* mutant cells. However, in succinate grown cells and in succinate plus glucose grown cells, trehalose and hexose-6-phosphates (likely fructose-6-phophate (6PS1), mannose-6-phosphate (6PS2) and glucose-6-phosphate (6PS3)) accumulated to higher levels in the *dme* and the *dme tme* mutant strains than in the wild type (*P* ≤ 0.05) (Fig. 1– note different scales for Y-axes). The polyamine putrescine was observed to accumulate to high levels in succinate and in succinate plus glucose grown *tme* mutant cells (Fig. 1). Both putrescine and trehalose are compatible solutes whose accumulation is often associated with cellular stress [20–25]. The fact the metabolic changes observed in succinate-grown cells were also observed in cells grown with succinate plus glucose suggests that these changes are directly related to succinate catabolism rather than an insufficient synthesis of glucose.

### TCA cycle intermediates accumulate in the DME mutant

**Fig. 2:**
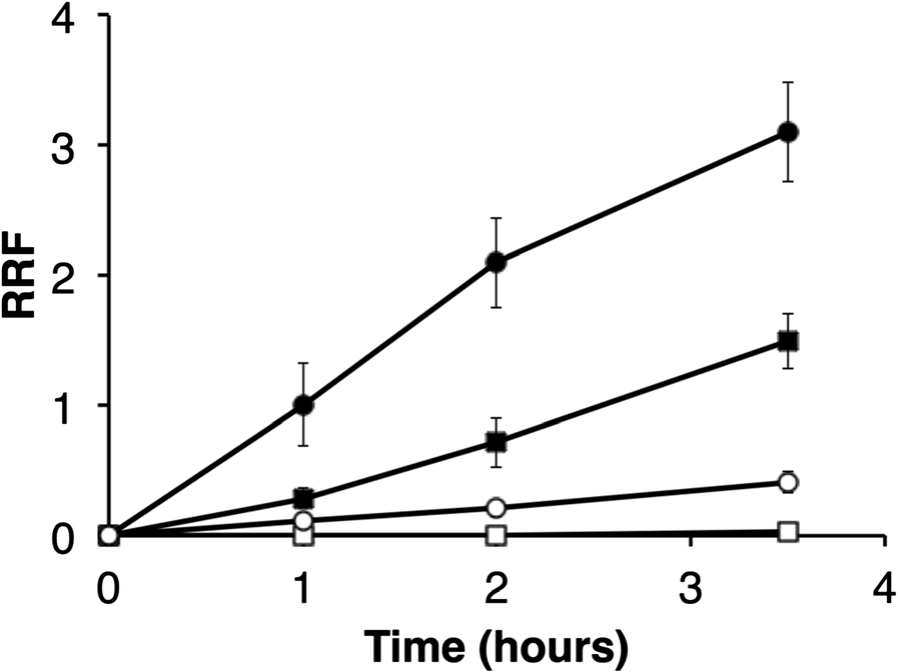
Excretion of malate (circles) and fumarate (squares) from *dme* (filled) and *tme* (open) mutant strains. Strains grown overnight in M9-glucose plus succinate were transferred into modified M9 containing 2.5 mM phosphate and 5 mM succinate. Samples were taken 1, 2 and 3.5 h post transfer and the culture supernatants were analyzed by GC-MS (see methods). The relative response factors for fumarate and malate relative to the standard ribitol were determined. Error bars were calculated using standard deviation of the means for three experimental replicates

As metabolites are often excreted from bacteria, we also analyzed the spent culture medium following growth of the various strains for the presence of extracellular polar metabolites. For these experiments, cells grown in regular M9 medium with glucose and succinate were centrifuged and re-incubated into a modified M9-medium containing succinate. Analysis of the supernatant over the ensuing 3.5 hour period revealed that malate and fumarate accumulated in the extracellular medium of *dme* mutant cells, whereas only a slight increase in the concentration of external malate and no fumarate was observed for the *tme* mutant (Fig. 2). After three hours of incubation in media containing 5 mM succinate, the levels of malate detected (see Methods) in the extracellular media of the *dme* mutant, the *tme* mutant, and wild-type strains were 613 ± 75 μM, 13 ± 3 μM, and 20 ± 9 μM respectively. At 613 μM, this represents ~12 % of the succinate originally present in the medium. The large amounts of malate, and fumarate, exported by the *dme* mutant suggests that these compounds, which serve as the substrate precursors for DME, accumulated in the mutant to high levels, presumably due to a decreased rate of conversion of malate to pyruvate.

However, the intracellular levels of malate and fumarate in succinate-grown *dme* and *tme* mutant cells were not significantly different (*p* < 0.05) (data not shown) presumably due to rapid export of accumulating metabolites. We therefore examined the intracellular profile of cells grown with succinate plus glucose as carbon sources. The GC-MS results showed that both malate and fumarate, as well as aspartate, accumulated to over twenty fold higher concentrations in *dme* cells than *tme* mutant cells (data not shown), suggesting that glucose addition exaggerates the build-up of TCA cycle intermediates.

### Global transcriptional analysis

Transcriptional effects were investigated using whole genome microarrays similar to those previously used to identify genes involved in hydroxyproline metabolism and cobalt transport in *S. meliloti* [26, 27]. Hierarchical cluster analyses of the transcriptional profiles showed that the transcriptomes clustered first based on the growth media (glucose vs succinate), and then separately by strain, with differences between the wild-type, *dme*, and *tme* strains most pronounced when grown with succinate (Additional file 1: Figure S1).

The differentially expressed genes were grouped according to their biochemical function and these changes are summarized in Table 1 (fold change ≥ 3 and *P* ≤ 0.05). Relatively few genes were differentially expressed in the *tme* mutant compared to the wild type in either glucose or succinate grown cells, and no clear patterns or specific pathways were detected. In contrast, 178 up-regulated and 19 down-regulated genes were detected in the succinate-grown *dme* mutant. Of the down-regulated genes, 16 were hypothetical, 2 encoded ribosomal proteins, and the last was a chemical resistance gene; as these lacked annotate function, they were uninformative with respect to the role of DME. On the other hand, only 26 % (47/179) of the up-regulated genes were hypothetical, while a further 30 % (54/179) are annotated as related to solute transport. To identify possible metabolic alterations, genes with annotated functions that were differentially expressed were mapped to their corresponding enzyme reactions in pathways for central carbon, and amino acid and fatty acid metabolism in *S. meliloti*.

**Table 1:** Overview of the differential expression patterns based on gene function categories^a^

Gene category	No. of genes significantly regulated^b^ (increased/decreased)				
	Glucose vs succinate (wt)^c^	*dme* vs wt (succinate)^d^	*tme* vs wt (succinate)^e^	*dme* vs wt (glucose)^f^	*tme* vs wt (glucose)^g^
Amino acid metabolism	3/0	17/0	4/0	3/2	2/0
Cofactor and vitamin metabolism	3/0	12/0	1/0	0/0	0/0
Fatty acid, ester, and phospholipid	1/1	4/0	2/0	0/0	1/0
Carbohydrate metabolism	13/3	13/0	2/0	0/0	0/0
Purine, pyrimidine, and nucleotide	0/1	1/0	3/0	1/0	1/1
Regulatory function	7/0	3/0	2/3	3/1	1/1
DNA replication and repair	1/1	4/0	1/1	0/0	0/1
Transport system	16/5	54/0	9/0	3/5	3/5
Energy metabolism	2/2	17/0	8/0	0/3	2/3
Other categories	4/3	7/3	3/2	2/2	5/0
Hypothetical protein	14/31	47/16	21/8	11/4	12/8
Total (6269)	64/47	179/19	56/14	23/17	27/19

^a^The complete list of differentially regulated genes can be found in Additional file 1: Tables S3–S6

^b^Genes significantly regulated have a fold change of ≥ 3 and a *P*-value (Student’s *t* test) of ≤ 0.05

^c^Comparing *S. meliloti* wild type grown in glucose and succinate

^d^Comparing *dme* mutant & wild type grown in succinate (includes *smb20178* and *smb2080* Additional file 1: Table S2)

^e^Comparing *tme* mutant and wild type grown in succinate

^f^Comparing *dme* mutant and wild type grown in glucose

^g^Comparing *tme* mutant and wild type grown in glucose

### Gluconeogenic flux is increased in the DME mutant

**Fig. 3:**
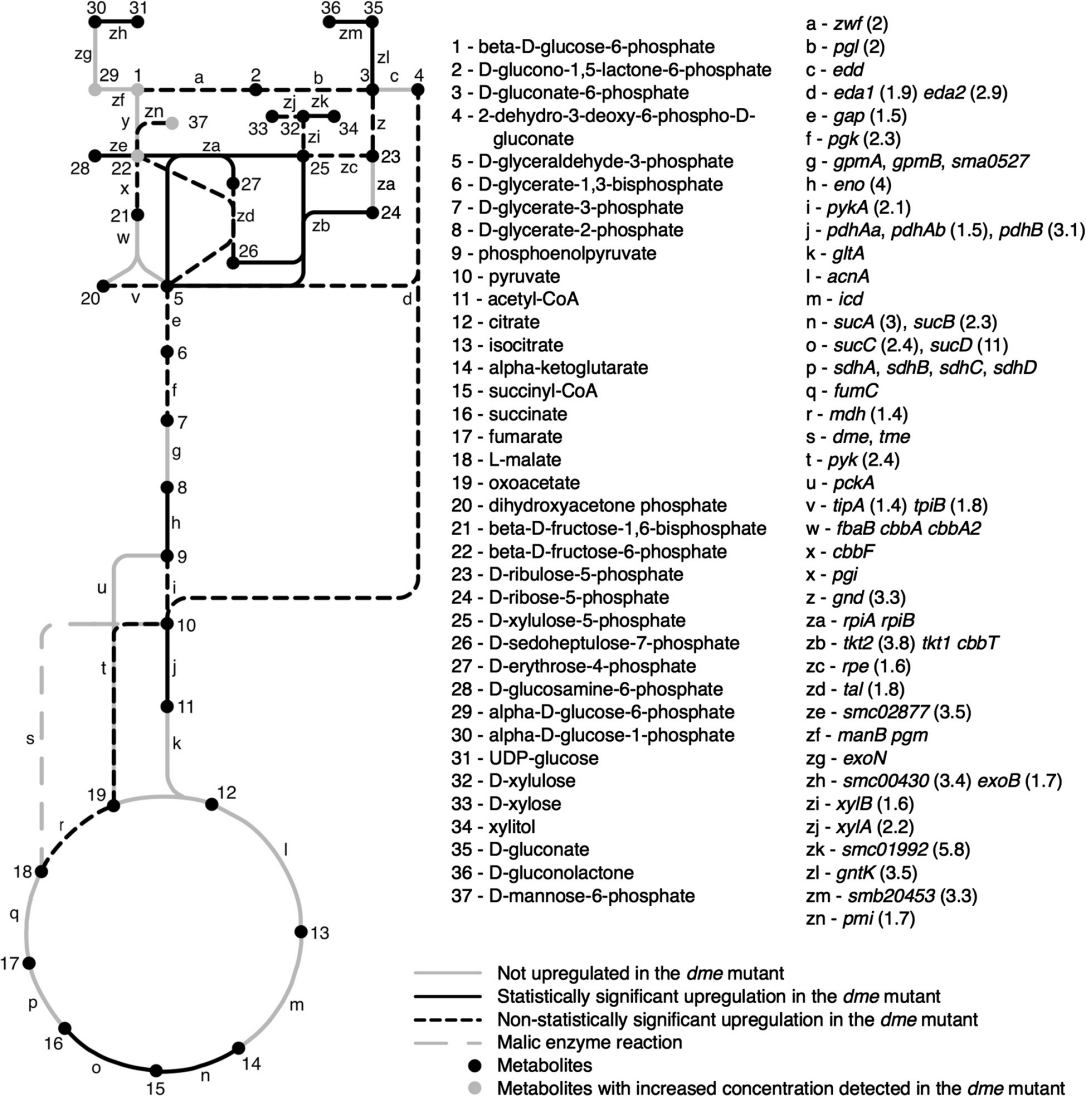
Schematic of genes and enzymes involved in central carbon metabolism in S*. meliloti*. Reactions are colour coded based on whether the corresponding genes were upregulated or not

Mapping of the genes expressed more highly in the *dme* mutant than the wild type to the pathways of central carbon metabolism indicated many genes related to sugar metabolism and inter-conversion of sugar metabolites (Fig. 3). This suggested a greater abundance of the corresponding precursor metabolites as a result of an increased gluconeogenic flux in the succinate-grown *dme* mutant than in the wild type. This is supported by the metabolite analysis, which identified hexose-6-phosphates as accumulating to higher levels in the *dme* and the *dme tme* mutant strains than in the wild type (Fig. 1). An accumulation of sugars in the *dme* mutant is further reinforced by the up-regulation of 14 solute transport genes annotated as putative sugars transporters, and among these, *smc04396* and *smb20036* are induced by dextrin and the cyclic polyol quinic acid, respectively [28]. The succinate-grown *dme* mutant cells also showed increased transcription of the TCA cycle genes *mdh* (malate dehydrogenase), *sucCD* (2-oxoglutarate dehydrogenase) and *sucAB* (succinyl-CoA synthetase) and this result is consistent with the co-transcription of these genes in the *mdh-sucCD-sucAB* operon [29]. Overall, these observations are consistent with an up-regulation of sugar synthesis to divert carbon away from the TCA cycle and reduce the build up of TCA cycle intermediates.

### Amino acid and fatty acid biosynthetic genes are up-regulated in the DME mutant

**Fig. 4:**
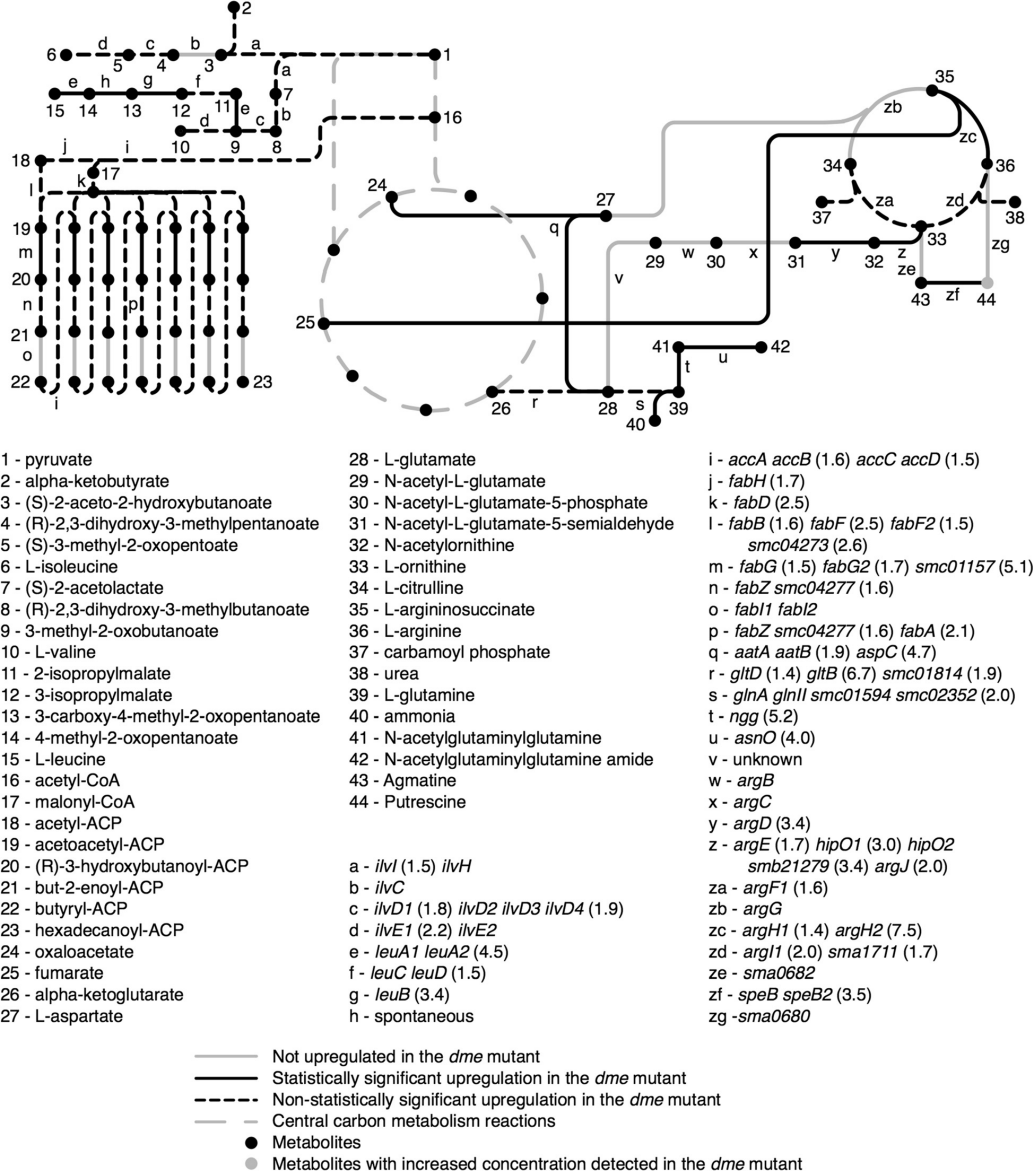
Schematic of genes and enzymes involved in amino acid and fatty acid synthesis in *S. meliloti*. Reactions are colour coded based on whether the corresponding genes were upregulated or not

Thirty-four amino acid biosynthetic genes were up-regulated in succinate-grown *dme* mutant cells compared to the wild type (Fig. 4). Many of these pathways employ pyruvate, fumarate, oxaloacetate, or 2-oxoglutarate as precursor compounds [30]. Additionally, 27 genes involved in the transport of amino-compounds such as spermidine/putrescine, peptides/oligopeptide, and amino acids were also highly induced in succinate-grown *dme* mutant cells. Examples include *smc03131, smc00671*, *sma0800*, and *smb20383* annotated as encoding histidine and spermidine/putrescine transport proteins. The gene *smc03131* was previously shown to be induced by DL-2-aminoadipic acid [28]. These observations are consistent with an increased synthesis of amino acids from central carbon metabolites in the *dme* mutant as a method to divert carbon away from the TCA cycle, and the subsequent excretion of these amino acids to the external environment where they induce their corresponding transporter.

Rather surprisingly, a number of genes annotated as involved in the biosynthesis of fatty acids from acetyl-CoA were also up-regulated in the succinate-grown *dme* mutant cells (Fig. 4). The physiological and genetic basis for this increased transcription is less clear, as one might expect that the levels of the fatty-acid precursor acetyl-CoA to be reduced in *dme* mutant cells relative to wild-type when utilizing succinate. However, fatty acid biosynthesis involves NADPH as an electron donor [1], and thus, the increased fatty acid biosynthesis may be a response to reduce a build-up of NADPH reductant in the *dme* mutant that results from increased pyruvate synthesis by the NADP^+^ dependent TME malic enzyme.

### DME mutations result in a growth phenotype with succinate

**Fig. 5:**
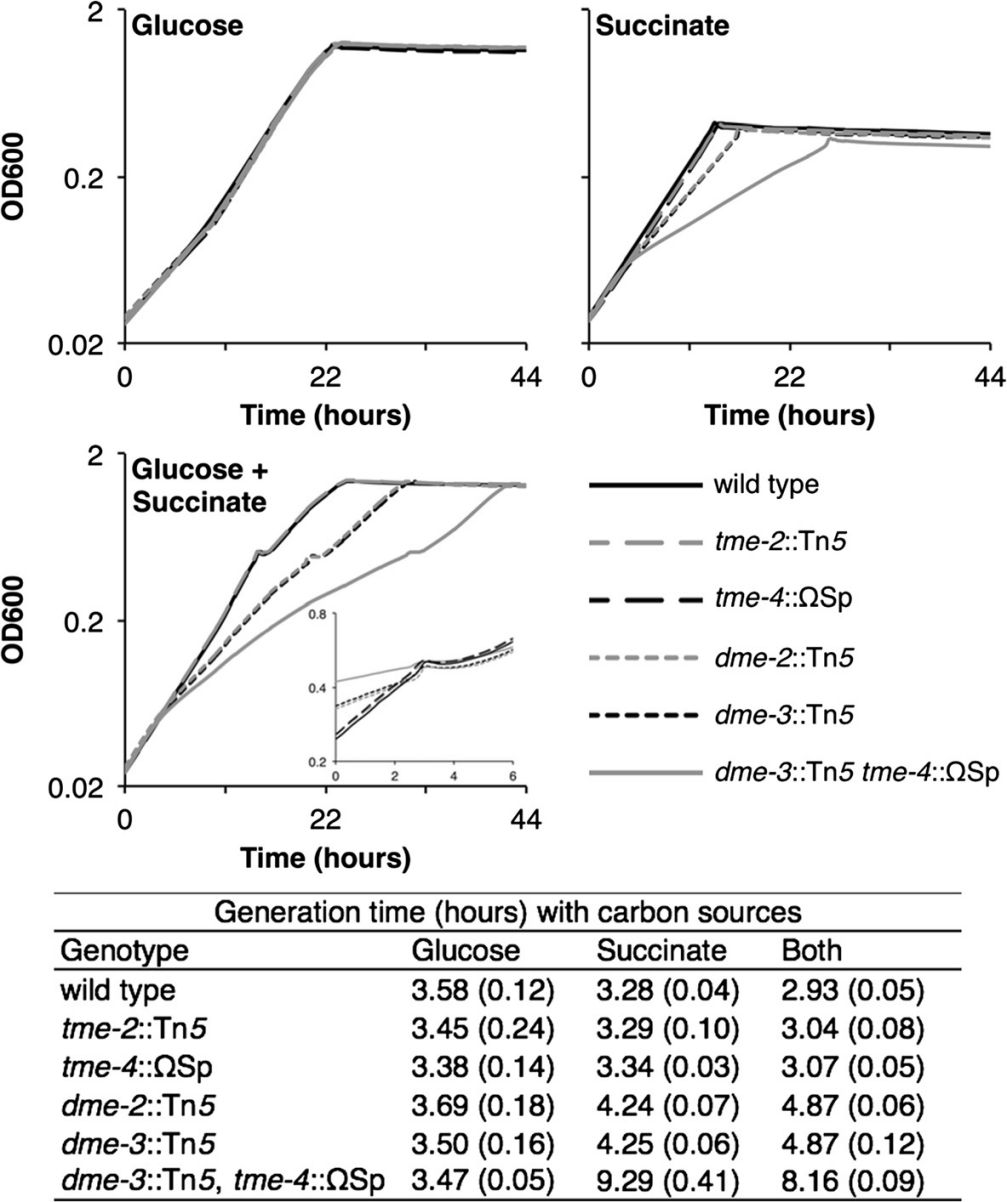
Growth of wild-type strain, the *dme*, the *tme* and *dme tme* double mutants grown in M9-Glucose (5 mM), M9-Succinate (5 mM), or M9-Succinate (5 mM) plus Glucose (5 mM). OD_600_ values were from triplicate samples. The insert in the glucose + succinate panel, shows OD_600_ values between 0.2 and 0.8 over the part of the growth curves where first growth stops and the second growth resumes. Note, that because the strains entered the diauxic growth transition at quite different times, the insert graph plots the OD_600_ from 3 h prior to end of first growth phase to 3 h past end of first phase, allowing all growth curves to be aligned at the end of the first growth phase. Generation times in hours were determined at culture densities between OD_600_ 0.1–0.3 (prior to the temporary halt in growth in the glucose + succinate cultures) and values are the means from the triplicate cultures +/− standard deviation of the mean. Both refers to media containing both succinate and glucose as carbon source

The metabolite and transcript analysis described above showed that growth of the malic enzyme mutants with succinate as the carbon source had greater effects than growth with glucose. This prompted us to perform a more detailed growth analysis of the wild-type, *dme*, *tme*, and *dme tme* double mutant strains (Fig. 5). The results revealed that the loss of TME had no effect on growth with succinate, or glucose, or glucose plus succinate. The loss of DME had no effect on growth with glucose alone but growth on succinate was reduced and surprisingly the addition of glucose with succinate exaggerated the growth reduction. The loss of both enzymes had no effect on growth with glucose but growth on succinate or succinate plus glucose was much reduced relative to single *dme* mutants. This result showed that in a *dme* mutant growing on succinate, the TME protein fulfills only part of malic enzyme metabolic requirement, and hence growth and metabolism is further reduced in the *dme tme* double mutant. These experiments also revealed a diauxic-like growth pattern for cultures growing in the media containing succinate plus glucose (see insert in Fig. 5). A similar diauxic growth was previously noted for another wild-type *S. meliloti* strain in media containing succinate plus glucose [31]. We note that the diauxic growth was observed for wild-type and all of the mutant strains (Fig. 5).

### Transcription and metabolite data

The comparison of the transcription data for wild-type with *dme* or *tme* data sets revealed transcriptional changes, some of which were quite modest (Additional file 1: Figure S1). Evidence that these transcription differences are robust include the following: a) genes encoding different steps in the same metabolic pathway show similar co-regulation, b) genes present in operons showed similar co-regulation patterns (*smc03978-81*) (Figs. 3 and 4), c) when *gusA* or *gfp* reporter gene fusions to nine different transcripts [32] were assayed in wild type, *dme*, and *tme* backgrounds, the resulting gene fusion data showed a very good correlation with the microarray values (Additional file 1: Table S1), d) the transcription patterns observed for well characterized genes such as *pckA*, *dctA*, *edd*, *dme*, and *tme* in glucose vs succinate-grown cells were the same as those previously observed in *S. meliloti* using microarrays [32] reporter gene fusions and enzymes assays [19, 33–37].

To investigate whether the observed metabolic alterations were influenced by particular mutant strains or culture conditions, we examined the metabolic profiles of different *dme* and *tme* mutant cells and also cells harvested at different culture densities. These experiments revealed that the metabolite changes shown in Fig. 1 for *dme* and *tme* mutants were robust as they were observed in cells harvested at different culture densities (OD_600_ of 0.2 and 0.8) and from cultures carrying *dme2* and *dme3* and *tme4* and *tme2* mutant alleles (data not shown).

## Discussion

Overall, greater effects were observed with the *dme* mutation compared to the *tme* mutation, and the effects of both mutations were greater in cells utilizing succinate compared to glucose as carbon source. Aside from putrescine accumulation in succinate-grown cells, no clear metabolomic or gene expression patterns were identified and no growth phenotype was observed in the *tme* mutant strains (Figs. 1 and 5). On the other hand, succinate-grown cells of the *dme* mutant accumulated hexose sugar phosphates and trehalose and excreted large amounts of malate and fumarate (Figs. 1 and 2). These cells also showed increased transcription of gluconeogenic genes (Fig. 3) and *dme* mutant also displayed a growth defect when grown with succinate and the presence of glucose in addition to succinate exaggerated this growth phenotype (Fig. 5). Together, these data strongly suggest that the DME enzyme of *S. meliloti* maintains metabolic homeostasis and balances TCA cycle flux in cells that are utilizing a gluconeogenic substrate such as succinate as a source of carbon. We conclude that succinate utilization in cells lacking DME leads to malate accumulation, and that malate is removed from the TCA cycle either through excretion and/or by diverting TCA cycle intermediates through alternative pathways (i.e., gluconeogenesis and amino acid and fatty acid biosynthesis).

The role for DME balancing the TCA cycle metabolite flux is consistent with previously reported allosteric activation of DME activity by succinate, fumarate, and malate, and its potent inhibition by acetyl-CoA [11]. DME and TME are present at similar activity levels in wild-type cells [13], and in concordance with previous observations, the transcript profile data revealed that the absence of one enzyme does not influence the expression of the other [10, 12]. The growth phenotype and metabolic data show that the active TME in *dme* mutant cells is insufficient to fully replace the physiological role of DME (Figs. 1 and 5). However, the physiological activity of TME does partially overlap with that of DME as *dme tme* double mutants had a reduced growth rate in the presence of succinate relative to single *dme* mutants (Fig. 5). This indicates that TME, and not PEP-carboxykinase and pyruvate kinase, serves as the main secondary pathway for pyruvate synthesis in succinate grown *S. meliloti*. These data are also consistent with previous suggestions that the role of TME is to maintain a basal malate to pyruvate flux that occurs even when DME is inhibited by high concentrations of acetyl-CoA [5, 10, 11].

The reduced growth phenotypes of free-living *dme* and the *dme tme* mutants with succinate (Fig. 5) appeared to be due to the accumulation of the TCA cycle intermediates, and not due to a lack of pyruvate. Pyruvate dehydrogenase (Pdh) is rapidly induced upon growth of *S. meliloti* on pyruvate [38], and because *dme* mutant cells grown with succinate showed a slight increase in transcription of genes encoding pyruvate kinase (Pyk) and Pdh relative to wild-type cells (Fig. 3), we suggest that the *dme* mutant cells synthesize substantial pyruvate. This would mean that the growth defect is likely not directly a consequence of a lack of pyruvate and a lack of ATP production, but rather a result of the accumulation of the TCA cycle intermediates. This hypothesis is supported by the observation that the reduced growth phenotype of succinate-grown *dme* and *dme tme* mutant strains is exaggerated if glucose is also present in the medium (Fig. 5); the presence of glucose may reduce the gluconeogenic flow of the mutant, increasing the accumulation of TCA cycle intermediates. The slow growth phenotype of the *dme* and *dme tme* mutants did not manifest itself until several hours following subculturing into succinate containing media (Fig. 5), perhaps as it takes several hours for the TCA cycle intermediates to reach inhibitory levels. It is also interesting to note that when grown with just succinate, the growth is logarithmic but slow, whereas in succinate + glucose, growth continuously gets slower. Thus, the glucose may be taken up slowly in the presence of succinate, and so does not have an effect early on, but as it is continuously taken up (and not metabolized), the growth continually gets poorer.

The diauxic growth phenotype observed in media containing succinate plus glucose is similar to the succinate mediated catabolite repression (SMCR) of lactose utilization that has been studied in some detail in *S. meliloti* [39]*.* While the mechanistic details and identity of the major physiological effector molecule(s) responsible for SMCR remain to be fully elucidated, the relative levels of phosphoenolpyruvate and glutamine have been suggested to be involved in SMCR signalling [40]. The *dme* or *tme* mutations did not appear to effect the levels of the SMCR signalling effector molecule(s) as the diauxic growth phenotype for *dme* and *tme* mutant strains in media containing succinate plus glucose was similar to the wild type (Fig. 5). While the metabolism of glucose by *Sinorhizobium* has been examined [41, 42], we are unaware of studies on the co-metabolism of succinate and glucose. Information on co-metabolism of these substrates, such as that available for *Bacillus subtilis* [43] would be valuable as while neither *dme* nor *tme* mutations effected the diauxic growth phenotype, the presence of glucose did effect the growth rate of the wild-type, *dme*, and *tme* strains on succinate (Fig. 5). These data suggest that there is some utilization of glucose during the first phase of growth (OD_600_ 0.02–0.3) in media containing succinate plus glucose.

The accumulation of trehalose and putrescine in the succinate-grown *dme*, and *tme* mutant cells, respectively, suggests that these cells are stressed (Fig. 1). We note that accumulation of trehalose (and hexose-6-phosphates) in the *dme* mutant maybe an indirect consequence caused by the increased gluconeogenic flux resulting from the diversion of metabolites from the TCA cycle. The levels of putrescine and other polyamines have been shown to respond to osmotic stress in other bacteria [20–22]. The elevated level of putrescine observed in succinate-grown *tme* mutant cells was dependent on DME as the level of putrescine in the *dme tme* double mutant was similar to wild-type (Fig. 1). Because cellular concentrations of both putrescine and trehalose can vary in response to cell stress [23–25, 44, 45], the reduced level of putrescine in the *tme dme* double mutant may be related to the elevated level of trehalose. Additionally, the genes *asnO* and *ngg* were 4.0 and 5.2 fold up-regulated in the succinate-grown *dme* mutant compared to the wildtype (Fig. 4). These two genes form a pathway for the synthesis of the compatible solute *N*-acetylglutaminylglutamine amide (NAGGN) in *S. meliloti* [46]. We observed no significant changes in *dme* versus wild type cells for the transcription of *otsA* or other genes implicated in trehalose synthesis in *S. meliloti* [*treY* (*smb20574*), *treZ* (*smb21447*), *treS* (*smb20099*)] [23, 24]. However in a related α-proteobacteria, *R. leguminosarum bv. trifolii*, trehalose accumulation appeared to be controlled by either post-transcriptional regulation or by control of breakdown rates [25]. Similarly no significant changes were observed for the transcription of genes with possible links in putrescine synthesis (*sma0680*, *sma0682, speB2*) [45] in *tme* versus wild type cells. However putrescine is a substrate for the homospermidine synthase (Hss) enzyme [46], and the *hss* gene was highly transcribed under all conditions examined. The levels of putrescine in *tme* cells could be controlled by effectors of homospermidine synthase. In summary, further studies are required to investigate the linkage between trehalose, putrescine, and the malic enzyme mutations.

Symbiotic N_2_-fixing bacteroids in root-nodules catabolise plant derived C_4_-dicarboxylic acids as their major source of carbon and energy, and DME is required for symbiotic N_2_-fixation in alfalfa nodules. While DME activity can be bypassed in free-living cells by alternate pathways (i.e., TME activity and the combined Pck and Pyk activities) Pck activity is not detected in N_2_-fixing *S. meliloti* bacteroids and can therefore not complement *dme* mutations [19]. The inability of TME to complement the loss of DME activity has remained an enduring question. The data reported here now provides insight into why this is the case: Nitrogenase has a high demand for ATP, requiring 16 moles of ATP per mole of fixed N_2_ [16]. In order to produce the required supply of ATP, the succinate and malate provided by the plant cell must be rapidly converted to pyruvate for energy production. As shown here, TME is unable to convert malate to pyruvate at the rate that this is performed by DME, and the differences between TME and DME may even be further amplified during symbiosis because the NADP in the bacteroid is predominantly reduced. Thus, the symbiotic phenotype of a *dme* mutant is likely a consequence of insufficient pyruvate synthesized by TME for nitrogenase activity. Additionally, as N_2_-fixing bacteroids are osmotically sensitive and mutations that effect osmotic sensitivity have been reported to affect symbiotic performance [47], there is a possibility that the accumulation of TCA-cycle intermediates and stress associated with the loss of DME activity might also affect symbiotic N_2_-fixation.

A comparison of the *S. meliloti dme* or *tme* mutant phenotypes with those of malic enzyme mutants of *E. coli* or *B. subtilis* revealed large differences. Loss of both enzymes from *E. coli* had no effect on growth with glucose or acetate; but the double mutant exhibited a much extended adaptive lag before growing on acetate [48]. The lag was attributed to a significant feedback interaction from metabolism to control of regulatory cAMP receptor protein (CRP) system. Of the four malic enzymes of *B. subtilis*, YtsJ reduces NADP^+^, whereas the three other enzymes reduce NAD^+^ (8.9). Mutations in these enzymes had no effect on growth with glucose or malate except for the NADP^+^-ME (*ytsJ*) mutant which grew poorly with malate. The authors suggested that YtsJ had a distinct function in the production of NADPH for biosynthetic purposes and that cells utilizing the gluconeogenic substrate malate do not produce sufficient NADPH by the pentose phosphate pathway. While these observations show that, not surprisingly, the phenotype of malic enzyme mutants is dependent on other pathways, it will be interesting to establish why the phenotype of NADP^+^-ME mutants of *E. coli* and *S. meliloti* is so different to that of *B. subtilis*.

## Conclusions

NAD(P) malic enzyme (DME) is shown to play an important role in maintaining TCA cycle flux and balanced levels of TCA cycle intermediates. In cells utilizing succinate, the loss of DME lead to the accumulation and excretion of malate, and an increased gluconeogenic flow that presumably resulted in the observed accumulation of hexose-6-phosphates and trehalose. The *dme* mutant cells had a reduced rate of growth on succinate and this was exacerbated upon the addition of glucose. Succinate-grown *tme* mutant cells accumulated putrescine and the causative link for this affect requires further investigation.

## Methods

### Bacterial strains and culture media

The genotype and source of the *S. meliloti* wild type and mutant strains used in this study are given in Table 2. With the exceptions of RmP2179 and RmP2189, all strains were previously described [10, 12]. To construct RmP2179, *tme*-4::ΩSp^r^/Sm^r^ was transduced from RmG994 to RmP110, and the absence of the TME protein in RmP2179 was verified by Western Blot analysis as described previously [13]. The *S. meliloti dme* mutant RmP2189 (RmP110, *dme-8*::ΩSp^r^/Sm^r^) was constructed by introducing the ΩSp^r^/Sm^r^ cassette [49] into the *dme* gene at nucleotide position 161 relative to the *dme* ATG start codon. The structure of the RmP2189 recombinant was verified by restriction analysis following PCR amplification of the *dme* region by use of primers (5'-GCTTCCTCGGTCACGACTTTC-3' and 5'-CTTCA TTTCTTCGTTGATGGTGC-3') from outside the region employed for mutant construction, and the absence of the DME protein was verified by Western Blot analysis [13]. Reporter gene fusions used to validate expression data were from the previously constructed *S. meliloti* pTH1522 gene fusion library [32] and were transferred to RmP110, RmP2189 and RmP2179. GusA and GFP assays were performed in triplicate as previously described [32].

**Table 2:** Primary *Sinorhizobium meliloti* strains used in this study

Strain	Relevant characteristics	Source
Rm1021	*S. meliloti* SU47, *str-21*	[12]
RmG454	Rm1021 *dme-2*::Tn*5*	[12]
RmG455	Rm1021 *dme-3*::Tn*5*	[12]
RmG456	Rm1021 *dme-1*::Tn*5*	[12]
RmG927	Rm1021 *tme-1*::Tn*5*	[10]
RmG994	Rm1021 *dme-3*::Tn*5 tme-4*::ΩSp^r^/Sm^r^	[10]
RmG995	Rm1021 *tme-4*::ΩSp^r^/Sm^r^	[10]
RmH215	Rm1021 *tme-2*::Tn*5*	[10]
RmP110	Rm1021 with changed wild-type *pstC*	[22]
RmP2179	RmP110 *tme-4*::ΩSp^r^/Sm^r^	This study
RmP2189	RmP110 *dme-8*::ΩSp^r^/Sm^r^	This study

*E. coli* were grown at 37 °C in Luria-Bertani broth (LB), while *S. meliloti* cells were grown at 30 °C in LBmc (LB supplemented with 2.5 mM MgSO_4_ and 2.5 mM CaCl_2_). The defined M9 minimal medium used to grow *S. meliloti* cultures for polar metabolite analysis contained 1× M9 salts (Difco) supplemented with 0.25 mM CaCl_2_, 1 mM MgSO_4_, 0.5 μg mL^-1^ biotin, and 43 nM CoCl_2_, with 5–15 mM succinate and/or glucose as the carbon source [26, 50]. MOPS (morpholinpropanesulfonic acid) buffered minimal medium used to grow *S. meliloti* cultures for microarray assay contains 40 mM MOPS, 20 mM KOH, 20 mM NH_4_Cl, 1.2 mM CaCl_2_, 100 mM NaCl, 2 mM MgSO_4_, 2 mM KH_2_PO_4_, 0.5 μg mL^-1^ biotin, and 1× trace element solution, with 15 mM succinate or 15 mM glucose as the carbon source. The trace element solution (1000×) consisted of the following amounts of compounds per liter of H_2_O: 1 g H_3_BO_3_, 1 g ZnSO_4_ · 7H_2_O, 0.5 g CuSO_4_ · 5H_2_O, 0.5 g MnCl_2_ · 4H_2_O, 1 g Na_2_MoO_4_ · 2H_2_O, 10 g Na_2_EDTA · 2H_2_O, and 2 g NaFeEDTA. Concentrations of antibiotics (μg mL^-1^) for *S. meliloti* strains were as follows: streptomycin (Sm), 200; gentamicin (Gm), 60; spectinomycin (Sp), 200; and neomycin (Nm), 200. For *E. coli* Gm and Sp were added at 10 and 100 μg/ml respectively.

### Microarray analysis

Microarray chips were purchased from NimbleGen Systems Inc., Madison,WI. RNA was extracted from 250 mL log phase cultures (OD_600_ of 0.4 to 0.8) of RmP110, RmP2179, and RmP2189 grown in MOPS-buffered minimal media with 15 mM succinate or 15 mM glucose. RNA extraction, cDNA end labelling with biotin, and hybridizations were done as previously described [26, 27]. The custom-made 4-plex arrays contained ~ 72 K probes of 60 oligonucleotides that targeted sequences within annotated start and end positions of 6269 annotated *S. meliloti* features, mostly protein-coding sequences but also some RNA sequences. Raw data probe intensities were quantile normalized across all experimental replicates (6 experiments × 2 replicates, or 12 arrays). Normalized probe intensities were subjected to a filter that required at least six non-redundant probes per feature. This reduced the number of features analysed to 6035. The median intensity of the pooled, filtered probes within an annotated region was used as an uncorrected measure of gene expression for each experiment. A set of 1786 null probes that did not match any *S. meliloti* genome sequence was used to estimate background for each array. Subtracting the array-estimated background from each feature median and averaging the two experimental replicates gave the average expression over each of the six experiments. Pair-wise experimental comparisons were made from the ratio of these background-corrected expression values and significance assessed using a Student’s *t*-test. The microarray data are available at Geo (http://www.ncbi.nlm.nih.gov/geo/) under the Accession Number GSE71308 and supplemental files (Additional file 1: Tables S2–S6 and Additional file 2: Dataset S1).

### Polar metabolite analysis

For each strain, three to five single colonies were grown overnight in LBmc and subcultured (1:200) into 50 mL of M9 containing 15 mM glucose or 15 mM succinate in a 250 mL flask. These cultures were incubated at 30 °C with shaking (200 rpm) and grown to the desired OD_600_ (0.2 to 0.8). Cells were harvested by centrifugation (20 min at 3730 *g*), resuspended in 1 mL of distilled water, and flash frozen in liquid nitrogen then stored at −80 °C. For extraction, ribitol and NaCl were added to thawed cells to final concentrations of 3 ng mL^-1^ and 29 mM, respectively. The cells were then transferred to screw-cap tubes containing 0.75 mL of 0.1 mm glass beads (Biospec products) and 400 μL of 100 % methanol, and were then lysed by bead beating for three 1 min periods in a Mini Beadbeater-8 with 1 min incubation on ice following each beating. Following centrifugation (5 min at 14,000 *g*) and transfer of the supernatant to a new tube, the beads were washed with 1 mL of 60 % methanol and then with 400 μL of 100 % methanol. 750 μL of 100 % chloroform was added to the resulting supernatant fraction, and following centrifugation (5 min at 14,000 *g*) the resulting aqueous phase (~1.75 mL) was lyophilized.

For analysis of extracellular metabolites, the high concentration of phosphate (69 mM) in regular M9 media interfered with the GC-MS analysis as the phosphate is derivatized by MSTFA and the trimethylsilylized phosphate molecules produced very large peaks that occluded surrounding peaks. To alleviate this issue, cultures grown in M9 containing 7.5 mM glucose and 7.5 mM succinate were harvested at an OD_600_ 0.5–0.6 and cells were centrifuged (20 min at 3730 *g*), washed with 0.85 % NaCl, and resuspended in a low phosphate minimal medium (20 mM NH_4_Cl, 2 mM MgSO_4_, 1.2 mM CaCl_2_, 100 mM NaCl, 0.5 μg mL^-1^ D-biotin, 0.01 μg mL^-1^ CoCl_2_, 2.5 mM KH_2_PO_4_ and 5 mM of the desired carbon source). Following incubation for 1–3.5 h, the cells were removed by centrifugation and 1 mL of each supernatant was flash frozen in liquid nitrogen and stored at −80 °C until needed. Ribitol was added to a final concentration of 3 ng mL^-1^, and prior to derivatization, the supernatant was filtered through Amicon Ultra Millipore columns to remove large molecular weight compounds (>10,000 Da). 200 μL of each sample was lyophilized overnight.

Methoxymation and silylation of metabolites were done using the procedures of Fiehn et al., [51] and Roessner et al. [52], and samples were run on a Trace GC 2000/Trace DSQ (Thermo Finigan, Gerstel) equipped with a Rtx-5MS Integra-Guard column (Restek) with helium carrier gas. Retention times for fatty acids standards and the response factor for the internal standard ribitol was recorded for each replicate [53, 54] and following a statistical analysis of the data with ANOVA, significant compounds (*P* ≤ 0.05) were identified by retention time using the NIST library (National Institute of Standards and Technology). Relative response factors (RRF) for all peaks were calculated by dividing the response factor of the peak by the response factor for ribitol. To validate the identity of trehalose and putrescine, a co-injection with a known amount of an authentic standard was performed.

The concentration of L-malate in the extracellular medium was quantified enzymatically using the K-MALAF kit from Megazyme International Ireland Limited. A standard curve for L-malate was constructed with known concentrations of L-malate in the growth media.

### Growth curves

Inoculum cultures grown in LBmc were washed once using carbon free M9 medium, then resuspended and diluted into M9 medium with the desired carbon source. Strains were grown in triplicate in 96-well microtitre plates in 150 μL cultures, and reported values are not corrected to a 1 cm pathlength. Growth conditions and analysis were as previously described [55].

## Abbreviations

DME, NAD(P)-malic enzyme; ED, Entner-Doudoroff; EMP, Embden-Meyerhof-Parnas; ETC, electron transport chain; Hss, homospermidine synthase; ME, malic enzyme; PDH, pyruvate dehydrogenase; PEP, phosphoenoylpyruvate; PTA, phosphotransacetylase enzyme; Pyk, pyruvate kinase; TCA, tricarboxylic acid; TME, NADP-malic enzyme

## Additional files

Additional file 1: Table S1. The relative expression levels of selected genes under different conditions determined by microarray and transcriptional fusion reporters. **Table S2.** Differentially expressed genes in *S. meliloti* free-living cells grown in MOPS (glucose) compared with MOPS (succinate). **Table S3.** Differentially expressed genes in succinate-grown cells of a *S. meliloti dme* mutant compared with the wild-type strain. **Table S4.** Differentially expressed genes in glucose-grown cells of a *S. meliloti dme* mutant compared with the wild-type strain. **Table S5.** Differentially expressed genes in succinate-grown cells of a *S. meliloti tme* mutant compared with the wild-type strain. **Table S6.** Differentially expressed genes in glucose-grown cells of a *S. meliloti tme* mutant compared with the wild-type strain. **Figure S1.** Hierarchical cluster analysis of the *S. meliloti* transcriptional profiles. (DOCX 117 kb) Additional file 2: Dataset S1. Background corrected microarray values and statistical analyses. The Excel file contains 5 sheets. All_Data: the complete set of background corrected microarray data. WT-DME-GLU: comparison between the glucose-grown wild type and *dme* mutant microarray data. WT-DME-SUC: comparison between the succinate-grown wild type and *dme* mutant microarray data. WT-TME-GLU: comparison between the glucose-grown wild type and *tme* mutant microarray data. WT-TME-SUC: comparison between the succinate-grown wild type and *tme* mutant microarray data. Column headings abbreviations: (W/D/T)(G/S)(1/2) – (wild type/*dme* mutant/*tme* mutant) (glucose grown/succinate grown) (replicate one / replicate two); -m – mean of the two replicates; -t – P-value as determined by a Student’s *T*-test; -fc – fold change in the mutant relative to the wild type. (XLSX 4642 kb)
