# Supplementary material for: Loss of malic enzymes leads to metabolic imbalance and altered levels of trehalose and putrescine in the bacterium Sinorhizobium meliloti
Source: BMC Microbiol. 2016 Jul 26;16:163. doi: 10.1186/s12866-016-0780-x (PMC4960864; doi:10.1186/s12866-016-0780-x)
Supplement: Additional file 1: Table S1. — The relative expression levels of selected genes under different conditions determined by microarray and transcriptional fusion reporters. Table S2. Differentially expressed genes in S. meliloti free-living cells grown in MOPS (glucose) compared with MOPS (succinate). Table S3. Differentially expressed genes in succinate-grown cells of a S. meliloti dme mutant compared with the wild-type strain. Table S4. Differentially expressed genes in glucose-grown cells of a S. meliloti dme mutant compared with the wild-type strain. Table S5. Differentially expressed genes in succinate-grown cells of a S. meliloti tme mutant compared with the wild-type strain. Table S6. Differentially expressed genes in glucose-grown cells of a S. meliloti tme mutant compared with the wild-type strain. Figure S1. Hierarchical cluster analysis of the S. meliloti transcriptional profiles. (DOCX 117 kb) [file 12866_2016_780_MOESM1_ESM.docx]

**Table S1.** The relative expression levels of selected genes under different conditions determined by microarray and transcriptional fusion reporters.

| Fusion strains | Gene ID | *dme/*wt (succinate)^b^ | |  | *tme*/wt (succinate)^c^ | |
| --- | --- | --- | --- | --- | --- | --- |
|  |  | Microarray | Fusion |  | Microarray | Fusion |
| SmFL5233 (*gfp*)^a^ | ***smc02047*** | NS | ND |  | 3.8* | 2.2* |
| SmFL4290 (*gfp*)^a^ | ***smb20171*** | 12.5* | 4.7* |  | 3.8* | 1.9* |
|  | *smb20172* | 10.4* |  |  | 3.1* |  |
| RmP2473 (*gusA*)^a^ | ***smb20173*** | 41.9* | 6.3* |  | 9.8* | 2.5* |
| SmFL6081 (*gusA*)^a^ | ***smb20174*** | 16.5* | 5.3* |  | 4.4* | 2.0* |
|  | *smb20175* | 17.8* |  |  | 6.6* |  |
| SmFL673 (*gusA*)^a^ | ***smb20204*** | 3.2* | 2.4* |  | NS | 0.9 |
|  | *smb20205* | 5.0* |  |  | NS |  |
|  | *smc20206* | 4.7* |  |  | 3.0* |  |
|  | *smb20207* | 3.8* |  |  | NS |  |
|  | *smb20208* | 4.9* |  |  | NS |  |
| RmP2479 (*gusA*)^a^ | ***smb20280*** | 3.4* | 2.2* |  | NS | ND |
| SmFL1445 (*gusA*)^a^ | ***smb20282*** | 3.0* | 2.8* |  | 2.1* | 1.2 |
| SmFL1913 (*gfp*)^a^ | ***smb20481*** | 4.0* | 2.2* |  | NS | ND |
| SmFL1896 (*gusA*)^a^ | ***smb21441*** | NS | ND |  | 1.8* | 1.1 |

Confirmation of the microarray data for select genes/operons. Transcriptional reporter gene fusions in wild-type, *dme*, and *tme* mutants were grown with 15 mM succinate and assayed in triplicate for GFP or GusA activity (32). Fold change values for the microarray and reporter enzyme activities are reported, and statistically significant changes (*P* ≤ 0.05 as calculated with a Student’s *t* test) are indicated with asterisks.

NS – changes in gene expression were not statistically significant.

ND – not determined.

^a^ The reporter gene (*gfp* or *gusA*) is indicated. Fusions are to the first gene in putative operons (indicated in boldface), and microarray data for all genes in the putative operons are shown.

^b^ Comparing the *dme* mutant and the wild type grown in succinate.

^c^ Comparing the *tme* mutant and the wild type grown in succinate.

**Table** **S2.** Differentially expressed genes in *S. meliloti* cells grown in MOPS (glucose) compared with MOPS (succinate).

| Gene ID | Gene name | | Production description | FC (glucose/succinate) | | |
| --- | --- | --- | --- | --- | --- | --- |
|  |  |  |  | wt | *dme* | *tme* |
| *smc04210* |  | | Hypothetical protein | 7.9 | 2 | 0.9 |
| *smc04031* | *pip2* | | proline iminopeptidase, EC:3.4.11.5 (metabolism of Arg and Pro) | 11.5 | 1.1 | 1.6 |
| *smc04005* | *pykA* | | pyruvate kinase, EC:2.7.1.40 | 4.1 | 2.7 | 2.5 |
| *smc03153* | *eda2* | | KDPG aldolase, EC:4.1.2.14 | 5.7 | 3 | 7.5 |
| *smc03070* | *zwf* | | glucose-6-phosphate 1-dehydrogenase, EC:1.1.1.49 | 5.5 | 2.8 | 3.2 |
| *smc03069* | *pgl* | | 6-phosphogluconolactonase, EC:3.1.1.31 | 4.4 | 2.2 | 1.9 |
| *smc03068* | *edd* | | phosphogluconate dehydratase | 4.8 | 3.4 | 4.4 |
| *smc03035* | *fliL* | | flagellar transmembrane protein (Cell motility) | 4.9 | 1.9 | 1.7 |
| *smc03012* | *cheD* | | Chemoreceptor glutamine deamidase (Bacterial chemotaxis and Cell motility) | 3 | 1.5 | 1.4 |
| *smc02864* | *moeB* | | molybdopterin biosynthesis protein (metabolism of cofactors and vitamins) | 10.5 | 0.8 | 2.9 |
| *smc02835* | *glk* | | Glucokinase | 2 | 0.7 | 2.2 |
| *smc02646* |  | | Putative glycerophosphoryl diester phosphodiesterase, EC:3.1.4.46 | 3 | 0.8 | 1.3 |
| *smc02607* | *soxD* | | Sarcosine oxidase subunit delta, EC:1.5.3.1 (metabolism of amino acid: Gly, Ser, and Thr ) | 13.8 | 1.5 | 0.8 |
| *smc02562* | *pckA* | | phosphoenolpyruvate carboxykinase , EC:4.1.1.49 (Gluconeogenesis) | -18.3 | -10.7 | -15.4 |
| *smc02043* | *eda1* | | KDPG aldolase, EC:4.1.2.14 | 3.6 | 1 | 1.9 |
| *smc02032* |  | | ATP-binding protein (putative simple sugar ABC transport system) | 5.3 | 1.7 | 10.4 |
| *smc01877* | *recN* | | DNA repair protein | 4.4 | 4.7 | 1.9 |
| *smc01861* | *murE* | | UDP-N-acetylmuramoylalanyl-D-glutamate--2,6-diaminopimelate ligase, EC:6.3.2.13 (Peptidoglycan biosynthesis) | 9.9 | 2 | 1.9 |
| *smc01456* |  | | Hypothetical protein (signal peptide) | 3.4 | 1.3 | 1.4 |
| *smc01412* |  | | Hypothetical protein | 3.4 | 0.7 | 1.5 |
| *smc01158* |  | | Hypothetical protein | 3.9 | 0.8 | 1.3 |
| *smc01032* | *pdhC* | | Dihydrolipoamide S-acetyltransferase | 3.4 | 2 | 3.9 |
| *smc01031* | *pdhB* | | pyruvate dehydrogenase subunit beta | 2.5 | 2.1 | 4.5 |
| *smc01030* | *pdhA* | | pyruvate dehydrogenase alpha2 subunit protein, | 3.3 | 2 | 2.9 |
| *smc00874* | *corA2* | | magnesium/cobalt transporter CorA | 3.4 | 0.7 | 0.9 |
| *smc00809* |  | | Hypothetical protein | -3.5 | -1.2 | -2.8 |
| *smc00800* |  | | Hypothetical protein | -3 | -1 | -3.3 |
| *smc00773* | *potI* | | putrescine permease (ABC transport system) | 3.5 | 1.3 | 2.6 |
| *smc00768* | *aceA* | | Isocitrate lyase, EC:4.1.3.1 (Glyoxylate and dicarboxylate metabolism) | -3.2 | -1.7 | -5.5 |
| *smc00457* |  | | hypothetical protein | 4.6 | 1.2 | 1.3 |
| *smc00371* |  | | Hypothetical protein | -3.5 | -1.6 | -2.3 |
| *smc00098* |  | | Transcriptional regulator | 5.5 | 0.6 | 1.5 |
| *smb21664* |  | | Hypothetical protein | -12.8 | -3.9 | -18.9 |
| *smb21535* |  | | LysR family transcriptional regulator | 4.2 | 0.9 | 1.9 |
| *smb21531* |  | | Hypothetical protein | 12.4 | 0.7 | 1.2 |
| *smb21510* |  | | plasmid stability protein | 4.3 | 0.6 | 3.9 |
| *smb21483* |  | | Hypothetical protein | -8.1 | -1.6 | -4.8 |
| *smb21474* | *fabG* | | 3-ketoacyl-ACP reductase, EC:1.1.1.100 (fatty acid biosynthesis) | -3.6 | -0.9 | -2.1 |
| *smb21473* |  | | Hypothetical protein | -3.2 | -0.7 | -2.3 |
| *smb21456* |  | | Hypothetical protein | -4.3 | -2.3 | -4.6 |
| *Continued on the following page* | | | | | | |
|  | |  |  |  |  |  |
|  | | | | | | |
| *Supplemental Table S2-Continued* | | | | | | |
| Gene ID | Gene name | | Production description | FC (glucose/succinate) | | |
|  |  |  |  | wt | *dme* | *tme* |
| *smb21454* |  | | Hypothetical protein | -3.2 | -0.9 | -2.9 |
| *smb21444* |  | | Osmotic sensory protein | -4.1 | -1.3 | -4.7 |
| *smb21442* |  | | hypothetical protein | -3.6 | -1.2 | -2.5 |
| *smb21441* |  | | Inosine-5′-monophosphate dehydrogenase, EC:1.1.1.205 (synthesis of GTP from IMP) | -3.3 | -1.3 | -1.9 |
| *smb21406* |  | | Hypothetical protein | -3.5 | -1 | -1.5 |
| *smb21395* |  | | Hypothetical protein | -4.2 | -0.8 | -1.4 |
| *smb21330* |  | | Hypothetical protein | -4.8 | -1.4 | -2.6 |
| *smb21314* | *wgdA* | | RTX toxins and secreted calcium-binding protein | 4.2 | 1.5 | 2 |
| *smb21313* | *wgeB* | | bifunctional glycosyltransferase (biosynthesis of polysaccharides) | 3.1 | 0.9 | 2.1 |
| *smb21226* |  | | Hypothetical protein | 4 | 0.8 | 0.7 |
| *smb21222* |  | | Transcriptional regulator | 4.5 | 1.6 | 1.4 |
| *smb21095* |  | | polar amino acid permease (putative ABC transport system) | 3.5 | 1.2 | 2.5 |
| *smb21094* | *argH2* | | argininosuccinate lyase, EC:4.3.2.1 (metabolism of Ala, Asp, Glu, Arg, and Pro ) | 3.1 | 0.8 | 4.3 |
| *smb20982* |  | | Hypothetical protein | 5.6 | 5.5 | 0.9 |
| *smb20930* |  | | ATP-binding protein (putative simple sugar uptake ABC transport system | 3.4 | 0.8 | 1.3 |
| *smb20916* |  | | Hypothetical protein | 5.1 | 1.2 | 1.5 |
| *smb20904* |  | | ATP-binding protein (sugar uptake ABC transporter system) | 11.6 | 9.7 | 16.5 |
| *smb20903* |  | | permease (sugar uptake ABC transporter system) | 28.4 | 11.2 | 14.1 |
| *smb20902* |  | | periplasmic solute-binding protein (sugar uptake ABC transporter system) | 30.3 | 7.4 | 21.7 |
| *smb20897* |  | | Hypothetical protein | 3.7 | 2.2 | 1.2 |
| *smb20630* |  | | ATP-binding protein (Putative multiple sugar uptake ABC transport system) | 3.3 | 0.9 | 1.4 |
| *smb20617* | *thiG* | | thiazole synthase (biosynthesis of thiamine) | 5 | 2.7 | 5.3 |
| *smb20615* | *thiC* | | thiamine biosynthesis protein | 5 | 3.4 | 4.6 |
| *smb20611* | *dctA* | | C4-dicarboxylate transporter | -98 | -139 | -72 |
| *smb20505* | *tfxG* | | Trifolitoxin immunity protein | 7.3 | 1.6 | 3.7 |
| *smb20465* |  | | Hypothetical protein | -3 | -1.1 | -2.6 |
| *smb20454* |  | | Hypothetical protein | -7 | -1.4 | -7.2 |
| *smb20391* |  | | Cellulose synthase catalytic, EC:2.4.1.12 (Starch and sucrose metabolism) | 3.3 | 0.9 | 4.2 |
| *smb20383* |  | | substrate-binding protein (putative spermidine/putrescine ABC transport system ) | 3.6 | 1.3 | 1.8 |
| *smb20381* |  | | spermidine/putrescine permease (putative ABC transport system ) | 4 | 1.5 | 1.8 |
| *smb20227* | *ndiA1* | | Putative nutrient deprivation-induced protein | -6.1 | -2.1 | -3.9 |
| *smb20086* |  | | hypothetical protein | -3.3 | -1.6 | -3.8 |
| *smb20074* |  | | Hypothetical protein | -5.2 | -1.2 | -2.3 |
| *smb20073* |  | | Oxidoreductase | -5.7 | -1.2 | -8.8 |
| *smb20072* |  | | myo-inositol induced periplasmic solute-binding protein (simple sugar ABC transport system) | -3.7 | -1.3 | -4.6 |
| *smb20065* |  | | Hypothetical protein | -3 | -1 | -2.1 |
| *sma5002* |  | | Hypothetical protein | -3.4 | -1.3 | -2.5 |
| *sma2406* | *rhbD* | | Rhizobactin siderophore biosynthesis protein ( iron uptake) | -3.8 | -2.2 | -3.7 |
| *sma2347* |  | | Hypothetical protein | 5.6 | 1.4 | 2.1 |
| *sma2297* |  | | hypothetical protein | -3.1 | -3.1 | -2.3 |
| *sma2071* |  | | Hypothetical protein | -3.8 | -1.1 | -2 |
| *Continued on the following page* | | | | | | |
|  | |  |  |  |  |  |
|  | |  |  |  |  |  |
| *Supplemental Table S2-Continued* | | | | | | |
| Gene ID | Gene name | | Production description | FC (glucose/succinate) | | |
|  |  |  |  | wt | *dme* | *tme* |
| *sma2059* |  | | Hypothetical protein | -3.6 | -1.6 | -6.8 |
| *sma1882* |  | | Transcriptional activator | 15 | 1.9 | 1.7 |
| *sma1500* |  | | Oxidoreductase | 3.7 | 1.4 | 1.9 |
| *sma1184* | *nosF* | | NosF ATPase (ABC-2 type transport system) | 3.6 | 1.8 | 0.9 |
| *sma1141* |  | | FNR/CRP family transcriptional regulator | 5.6 | 1.7 | 1.6 |
| *sma1138* |  | | Hypothetical protein | 3 | 1.5 | 1.8 |
| *sma1050* |  | | Hypothetical protein | 3.3 | 1.5 | 2.7 |
| *sma0952* |  | | Mannopine Permease (ABC transport system) | 3.3 | 1.7 | 1.6 |
| *sma0849* | *syrM* | | SyrM transcriptional regulator | 3 | 1.5 | 2.5 |
| *sma0599* |  | | hypothetical protein | 6.7 | 1.6 | 1 |
| *sma0594* |  | | hypothetical protein | 5.4 | 1.6 | 1.9 |
| *sma0563* |  | | aldehyde or keto oxidase | 3.3 | 0.9 | 1.2 |
| *sma0541* |  | | Hypothetical protein | -3 | -1.4 | -2.3 |
| *sma0197* |  | | simple sugar permease (putative ABC transport system) | 4.2 | 10.9 | 12.3 |
| *sma0187* |  | | Short chain dehydrogenase | -3.1 | -1.8 | -2.1 |
| *sma0078* |  | | LacI family transcriptional regulator | 3.5 | 0.8 | 2.2 |
| *sma0067* |  | | periplasmic solute-binding protein (putative simple sugar ABC transport system) | 3.6 | 1 | 1.5 |

Genes are grouped by replicon and ordered by descending gene number. FC is fold change of gene expression (*S. meliloti* cultures grown in glucose vs succinate). Significantly regulated genes: FC ≥ 3 and *P*-value (Student’s *t* test) ≤ 0.05. Minus (-) genes are significantly downregulated. wt – *S. meliloti* wild type strains RmP110; *dme* – *S. meliloti dme* mutant RmP2189; *tme* – *S. meliloti tme* mutant RmP218

**Table S3.** Differentially expressed genes in succinate-grown cells of a *S. meliloti dme* mutant compared with the wild-type strain.

| **Gene ID** | **Gene name** | **Product description (functions)** | **FC**  **(*dme/*wt)** |
| --- | --- | --- | --- |
| SMc00078 | *livJ* | periplasmic SBP (branched-chain amino acid ABC transport) | 3.3 |
| SMc00385 |  | Hypothetical protein | 3 |
| SMc00389 |  | Hypothetical protein | 3.2 |
| SMc00430 |  | Putative UDP-glucose 4-epimerase, EC:5.1.3.2 | 3.4 |
| SMc00433 | *iolE* | Putative inosose dehydratase, EC:4.2.1.44 (myo-inositol metab) | 3.3 |
| SMc00671 | *hisW* | histidine permease (amino acid ABC transport) | 4.7 |
| SMc00696 | *aroB* | 3-dehydroquinate synthase, EC:4.2.3.4 | 3.4 |
| SMc00706 |  | hypothetical protein | 6.9 |
| SMc00769 |  | hypothetical protein | 5.8 |
| SMc00771 | *potG* | ATP-binding protein (putrescine ABC transporter) | 3.3 |
| SMc00788 | *dppC1* | dipeptide permeas ( ABC transport system ) | 3.6 |
| SMc00923 | *tag* | DNA-3-methyladenine DNA glycosylase, EC:3.2.2.20 | 3.8 |
| SMc01028 | *eno* | phosphopyruvate hydratase, EC:4.2.1.11 | 4 |
| SMc01032 | *pdhC* | Dihydrolipoamide S-acetyltransferase, EC:2.3.1.12 | 3 |
| SMc01036 |  | hypothetical protein | 4.7 |
| SMc01157 |  | Putative [acyl-carrier-protein] reductase, EC:1.1.1.100 | 5.1 |
| SMc01167 | *dnaA* | chromosomal replication initiation protein ( DNA replicate) | 3.1 |
| SMc01274 | *crcB* | hypothetical protein (signal peptide) | 3.1 |
| SMc01317 | *rpoB* | DNA-directed RNA polymerase, EC:2.7.7.6 | 4.6 |
| SMc01340 |  | aminotransferase | 3.6 |
| SMc01424 | *nthA* | nitrile hydratase alpha protein, EC:4.2.1.84 | 3.6 |
| SMc01469 | *mcpW* | Probable methyl accepting chemotaxis transmembrane protein | 3.4 |
| SMc01526 | *dppB2* | peptide/nickel permease (ABC transport) | 3.8 |
| SMc01574 |  | hypothetical protein | 3.1 |
| SMc01628 |  | periplasmic SBP (multiple sugar ABC transport) | 3.8 |
| SMc01639 |  | acyl-CoA dehydrogenase, EC:1.3.99.3 (Metab of fatty acid) | 6.9 |
| SMc01658 |  | Ferrioxamine B specific ferric iron reductase (iron uptake) | 3.5 |
| SMc01660 |  | hypothetical protein | 3.2 |
| SMc01779 |  | MFS-type transporter | 4.8 |
| SMc01782 |  | hypothetical protein | 3 |
| SMc01793 |  | glycosyltransferase (biosynthesis of polysaccharides) | 3.2 |
| SMc01820 |  | hypothetical protein | 3 |
| SMc01946 | *livK* | leucine-specific binding protein | 3.4 |
| SMc01947 |  | hypothetical protein | 3.4 |
| SMc01950 | *livM* | high-affinity branched-chain amino acid permease ( ABC system) | 3.4 |
| SMc01965 |  | ATP-binding protein (spermidine/putrescine ABC transport) | 3.1 |
| SMc01967 | *speB2* | agmatinase, EC:3.5.3.11 (conversion of agmatine to putrescine) | 3.5 |
| SMc01992 |  | D-xylulose reductase, EC:1.1.1.9 | 5.8 |
| SMc02103 |  | hypothetical protein | 3.8 |
| SMc02120 | *aapM* | general L-amino acid permease (ABC transport) | 3.3 |
| SMc02138 | *argD* | acetylornithine transaminase, EC:2.6.1.17 &2.6.1.11 | 3.7 |
| SMc02229 |  | acyl-CoA dehydrogenase, EC:1.3.99.- ( fatty acid metabolism) | 3.4 |
| SMc02262 | *aspC* | Aspartate transaminase, EC: 2.6.1.1 | 4.7 |
| SMc02305 | *murA* | UDP-N-acetylglucosamine 1-carboxyvinyltransferase, EC:2.5.1.7 | 3 |
| SMc02319 |  | hypothetical protein | 3.2 |
| SMc02350 |  | hypothetical protein | 3.1 |
| SMc02356 |  | periplasmic SBP (branched-chain amino acid ABC transport) | 5.7 |
| SMc02384 |  | glycosyltransferase transmembrane protein (synth of polysac) | 4.6 |
| SMc02469 |  | oxidoreductase | 4 |
| SMc02481 | *sucD* | succinyl-CoA synthetase subunit alpha, EC:6.2.1.5 (TCA cycle) | 10.9 |
| SMc02482 | *sucA* | 2-oxoglutarate dehydrogenase E1 component, EC:1.2.4.2 (TCA cycle) | 3 |
| SMc02546 | *leuA2* | Putative2-isopropylmalate synthase, EC:2.3.3.13 | 4.5 |
| SMc02549 |  | hypothetical protein | 3.1 |
| SMc02610 | *glxB* | Putative glutamine amidotransferase | 3.8 |
| SMc02653 | *lepB* | signal peptidase I transmembrane protein, EC:3.4.21.89 | 3.1 |
| SMc02684 |  | hypothetical protein | 3.3 |
| SMc02738 | *choW* | glycine betaine/proline permease (ABC transport) | 5.1 |
| SMc02758 |  | Putative nucleotidyl transferase (DNA repair) | 3.3 |
| SMc02825 | *pepA2* | Aminopeptidase (glutathione metabolism) | 3 |
| SMc02877 |  | Putative sugar isomerase (sugar metabolism ) | 3.5 |
| SMc02907 |  | amino acid efflux transmembrane protein | 4.2 |
| SMc03035 | *fliL* | flagellar transmembrane protein (cell mobility) | 4.2 |
| SMc03061 | *aglE* | Periplasmic SBP (alpha-glucoside ABC transport) | 4.2 |
| SMc03131 |  | periplasmic SBP (polar amino acid ABC transport) | 13.8 |
| SMc03162 |  | hypothetical protein | 3.1 |
| SMc03207 |  | hypothetical protein | 3.3 |
| SMc03822 |  | hypothetical protein | 4.3 |
| SMc03829 |  | putative ABC transport permease | 3.1 |
| SMc03931 | *soxA2* | sarcosine oxidase alpha, EC:1.5.3.1 | 3 |
| SMc03938 | *pntB* | NAD(P) transhydrogenase subunit beta (recyc NAD+ & NADP+) | 4.8 |
| SMc03939 | *pntAb* | NAD(P) transhydrogenase subunit alpha, EC:1.6.1.1 | 5.3 |
| SMc03943 |  | hypothetical protein | 3 |
| SMc03950 | *pntAa* | NAD(P) transhydrogenase subunit alpha, EC:1.6.1.1 | 3.2 |
| SMc03978 | *tkt2* | transketolase, EC:2.2.1.1 (Pentose phosphate pathway) | 3.8 |
| SMc04048 |  | cytochrome c protein ( electron transport ) | 3.2 |
| SMc04093 | *acsA1* | acetyl-CoA synthetase (conversion of acetate to acetyl-coA) | 3.2 |
| SMc04264 |  | transcriptional regulator | 5.1 |
| SMc04282 | *cobB* | cobyrinic acid a,c-diamide synthase ( Porphyrin and B12 metabolism ) | 4.1 |
| SMc04305 | *cobU* | adenosylcobinamide kinase/adenosylcobinamide-Pi guanylyltransferase | 3.6 |
| SMc04396 |  | periplasmic solute-binding protein (multiple sugar ABC transport) | 5.4 |
| SMc04405 | *leuB* | 3-isopropylmalate dehydrogenase, EC:1.1.1.85 | 3.4 |
| SMb20003 |  | pyrroline-5-carboxylate reductase, EC:1.5.1.2 | 4 |
| SMb20006 |  | hypothetical protein | 3.2 |
| SMb20036 |  | periplasmic SBP (Putative TRAP-type quinic acid transport) | 7.6 |
| SMb20057 | *cbtK* | Permease protein (Cobalt ABC transport system) | 9 |
| SMb20071 |  | efflux protein | 3.6 |
| SMb20107 |  | hypothetical protein | 4.3 |
| SMb20108 |  | periplasmic SBP (peptide/nickel ABC transport) | 3.1 |
| SMb20165 |  | hypothetical protein | 5.5 |
| SMb20166 |  | hypothetical protein | 4.8 |
| SMb20169 |  | hypothetical protein | 4.6 |
| SMb20171 |  | Putative S-formylglutathione hydrolase, EC:3.1.2.12 | 12.5 |
| SMb20172 |  | cytochrome c protein | 10.4 |
| SMb20173 |  | methanol dehydrogenase large subunit | 41.9 |
| SMb20174 |  | cytochrome c protein | 16.5 |
| SMb20175 |  | hypothetical protein | 17.8 |
| SMb20177 |  | hypothetical protein | 7.2 |
| SMb20178* |  | hypothetical protein | 2.4 |
| SMb20179 |  | hypothetical protein | 4.9 |
| SMb20180* |  | hypothetical protein | 2.2 |
| SMb20186 | *gfa* | Glutathione-dependent formaldehyde-activating enzyme | 12.2 |
| SMb20204 | *pqqA* | Pyrroloquinoline quinone(PQQ) synthesis | 3.2 |
| SMb20205 | *pqqB* | PQQ biosynthesis protein | 5 |
| SMb20206 | *pqqC* | PQQ biosynthesis protein | 4.7 |
| SMb20207 | *pqqD* | PQQ biosynthesis protein | 3.8 |
| SMb20208 | *pqqE* | PQQ biosynthesis protein | 4.9 |
| SMb20262 |  | NADP-semialdehyde dehydrogenase, EC:1.2.1.4 | 3.5 |
| SMb20280 |  | hypothetical protein | 3.4 |
| SMb20282 |  | permease protein (putative spermidine/putrescine ABC transport) | 3 |
| SMb20322 |  | permease protein (putative hydroxyproline trap-type transport) | 6.2 |
| SMb20342 |  | isoquinoline 1-oxidoreductase, EC:1.3.99.16 | 3.6 |
| SMb20365 |  | periplasmic SBP (iron(III) ABC transport ) | 6 |
| SMb20373 |  | permease protein (putative TRAP-type dicarboxylate transport) | 6.4 |
| SMb20374 |  | periplasmic SBP (putative TRAP-type dicarboxylate transport) | 5.1 |
| SMb20381 |  | permease protein (putative spermidine/putrescine ABC transport) | 3.5 |
| SMb20383 |  | periplasmic SBP (putative spermidine/putrescine transport) | 6.6 |
| SMb20385 |  | hypothetical protein | 5.5 |
| SMb20429 | *ehuC* | permease protein (putative polar amino acid ABC transport) | 9.2 |
| SMb20453 |  | gluconolactonase, EC:3.1.1.17 (Pentose phosphate pathway) | 3.3 |
| SMb20481 | *asnO* | asparagine synthetase, EC:6.3.5.4 (Ala, Asp, and Glu metabolism) | 4 |
| SMb20484 | *supA* | periplasmic sugar-binding protein (simple sugar ABC transport) | 3.6 |
| SMb20616 | *thiO* | thiamine biosynthesis oxidoreductase (thiamine metabolism) | 3.3 |
| SMb20617 | *thiG* | thiazole synthase (thiamine metabolism) | 3.8 |
| SMb20620 |  | SBP (simple sugar ABC transport system) | 3.1 |
| SMb20708 |  | methylated-DNA--protein-cys methyltrans (repair DNA) | 11.8 |
| SMb20865 |  | hypothetical protein | 3.5 |
| SMb20890 | *araF* | dihydroxy-acid dehydratase, EC:4.2.1.9 (Ara catabolism) | 3.1 |
| SMb20891 | *araE* | 2-oxoglutarate semialdehyde DH, EC 1.2.1.26 (Ara catabolism) | 3.8 |
| SMb20902 |  | periplasmic solute-binding protein (simple sugar ABC transport system) | 4 |
| SMb20930 |  | ATP-binding protein (putative simple sugar ABC transport system) | 3.3 |
| SMb21000 |  | putative transport protein | 5.5 |
| SMb21006 |  | hypothetical protein | 3.1 |
| SMb21049 |  | hypothetical protein | 4.4 |
| SMb21094 | *argH2* | argininosuccinate lyase, EC:4.3.2.1 (Ala, Asp, and Glu metabolism) | 7.5 |
| SMb21111 |  | oxidoreductase | 3.5 |
| SMb21119 | *gntK* | gluconokinase, EC:2.7.1.12 (Pentose phosphate pathway) | 3.5 |
| SMb21181 |  | glutaryl-CoA dehydrogenase, EC:1.3.99.7 | 3.2 |
| SMb21182 |  | Hypothetical protein | 10.5 |
| SMb21198 |  | permease protein (oligopeptid ABC transport system ) | 3.7 |
| SMb21206 |  | ATP-binding protein (ABC-2 type transport system for export) | 4.5 |
| SMb21221 |  | periplasmic SBP (Putative multiple sugar ABC transport system) | 3.8 |
| SMb21226 |  | hypothetical protein | 4.8 |
| SMb21275 |  | permease protein (putative spermidine/putrescine ABC transport) | 4.6 |
| SMb21333 |  | hypothetical protein | 3.6 |
| SMb21420 |  | L-arabinose isomerase (sugar metabolism) | 3.1 |
| SMb21424 |  | Putative acyl esterase (metabolism of ester) | 3.2 |
| SMb21461 |  | periplasmic SBP (putative trehalose/maltose ABC transport) | 3.2 |
| SMb21475 |  | hypothetical protein | 4.3 |
| SMb21499 |  | hypothetical protein | 4.7 |
| SMb21535 |  | LysR family transcriptional regulator | 4.7 |
| SMb21550 |  | hypothetical protein | 3 |
| SMb21558 |  | aldehyde or xanthine dehydrogenase, iron-sulfur subunit protein | 5.8 |
| SMb21691 |  | Alkanesulfonate monooxygenase (amino acid metabolism) | 3.9 |
| SMa0009 | *fdhE* | formate dehydrogenase accessory protein | 3 |
| SMa0067 |  | periplasmic solute-binding protein (simple sugar ABC transport system) | 4.1 |
| SMa0070 |  | simple sugar permease ( ABC transport system) | 27.5 |
| SMa0087 |  | hypothetical protein | 3.3 |
| SMa0105 |  | permease protein (Peptides/nickel ABC transport system) | 3.3 |
| SMa0237 |  | dehydrogenase | 3.8 |
| SMa0396 |  | permease protein (spermidine/putrescine ABC transport system) | 6.2 |
| SMa0525 |  | permease protein (iron(III) ABC transport system ) | 3.8 |
| SMa0800 |  | permease protein (spermidine/putrescine ABC transport system) | 5 |
| SMa1168 |  | Dehydrogenase, FAD-dependent | 4.3 |
| SMa1259 |  | hypothetical protein | 3.2 |
| SMa1294 |  | hypothetical protein | 5.3 |
| SMa1406 | *ttuD3* | hydroxypyruvate reductase, EC:1.1.1.81 (Gly, Ser and Thr) | 15.4 |
| SMa1410 |  | Oxidoreductase | 8.3 |
| SMa1500 |  | oxidoreductase | 4.2 |
| SMa1650 |  | permease protein (peptide/nickel ABC transport) | 7.7 |
| SMa1664 |  | HlyD-family protein (ABC export system) | 4.3 |
| SMa1787 |  | transposase, fragment | 3.1 |
| SMa1817 |  | hypothetical protein | 3.4 |
| SMa1863 |  | permease protein (Peptides/nickel ABC transport) | 3.7 |
| SMa1882 |  | transcriptional activator | 5 |
| SMa1913 |  | Na+/H+ antiporter (Methane metabolism) | 4.2 |
| SMa2087 |  | Desulfurization enzyme | 3.4 |
| SMa2117 |  | oxidoreductase | 3.2 |
| SMa2119 |  | hypothetical protein | 3.2 |
| SMa2203 |  | permease protein ( putative spermidine/putrescine ABC transport) | 3.3 |
| SMa2361 |  | hypothetical protein | 4.9 |
| SMa5034 |  | hypothetical protein | 3.5 |
|  |  |  |  |
| SMc00323 | *rpsO* | 30S ribosomal protein S15 | -3 |
| SMc00809 |  | hypothetical protein | -3.2 |
| SMc01791 |  | hypothetical protein | -4 |
| SMc01369 | *rpmG* | 50S ribosomal protein L33 | -3 |
| SMc00252 |  | hypothetical protein | -4.1 |
| SMc00198 |  | hypothetical protein | -3.4 |
| SMc04184 |  | hypothetical protein | -4.6 |
| SMc04186 |  | hypothetical protein | -3.5 |
| SMc04194 |  | hypothetical protein | -3.1 |
| SMc01551 |  | hypothetical protein | -3 |
| SMc01586 |  | hypothetical protein | -3.2 |
| SMc01523 | *emrE* | Putative methyl viologen/ethidium resistance | -3.8 |
| SMa1678 |  | hypothetical protein | -3.1 |
| SM_b22008 |  | hypothetical protein | -3 |
| SMb21329 |  | hypothetical protein | -3.1 |
| SMb21574 |  | hypothetical protein | -4.4 |
| SMb21685 |  | hypothetical protein | -4.5 |
| SMb21456 |  | hypothetical protein | -3.8 |
| SMb20711 |  | hypothetical protein | -5.5 |
|  |  |  |  |

Genes are grouped by replicon and ordered by descending gene number. FC (*dme/*wt) is the fold change of gene expression (*S. meliloti* succinate-grown cells of the *dme* mutant vs wild type). Significantly regulated genes: FC ≥ 3 and *P*-value (Student’s *t* test) ≤ 0.05. Minus (-) genes are significantly downregulated. wt – *S. meliloti* wild type strains RmP110; *dme* – *S. meliloti dme* mutant RmP2189.

* *smb20178** and *smb2080** are included as they are part of the *smb20178-smb20185* operon-like gene cluster.

**Table S4.** Differentially expressed genes in glucose-grown cells of a *S. meliloti dme* mutant compared with the wild-type strain.

| Gene ID | Gene name | Production description (functions) | FC (*dme*/wt) |
| --- | --- | --- | --- |
|  |  |  |  |
| *smc04220* |  | transcriptional regulator | 13 |
| *smc04028* | *gltB* | glutamate synthase, (biosynthesis of Glu from 2-oxoglutarate) | 3.9 |
| *smc03107* |  | hypothetical protein | -16.5 |
| *smc02525* | *fdsB* | NAD-formate dehydrogenase subunit beta, (Methonal metabolism) | -3.7 |
| *smc02372* |  | Putative MFS permease | -3.7 |
| *smc02180* |  | hypothetical protein | -3.1 |
| *smc02035* |  | Oxidoreductase | -3.7 |
| *smc02028* |  | permease protein (peptide/nickel ABC transport system ) | -4.2 |
| *smc01626* |  | permease protein (putative multiple sugar ABC transport) | 3.5 |
| *smc01509* |  | hypothetical protein | 5.2 |
| *smc00703* |  | hypothetical protein | 3.1 |
| *smc00682* | *hipO1* | hippurate hydrolase, EC:3.5.1.32 (Phe metabolism) | 3.1 |
| *smc00371* |  | hypothetical protein | 3 |
| *smc00259* |  | hypothetical protein | 3.9 |
| *smb21633* | *paaG* | Enoyl-CoA hydratase, (metabolism of Phe to acetyl-CoA ) | -3.1 |
| *smb21510* |  | Putative plasmid stability protein | -7.8 |
| *smb21509* |  | Putative plasmid stability protein | -6.1 |
| *smb21483* |  | hypothetical protein | 3 |
| *smb21442* |  | hypothetical protein | 3.4 |
| *smb21441* |  | Inosine-5′-monophosphate dehydrogenase, (synthesis of GTP from IMP) | 3.6 |
| *smb21395* |  | hypothetical protein | 4.3 |
| *smb21162* |  | Putative MFS permease | -5.7 |
| *smb21154* |  | hypothetical protein | -4.8 |
| *smb21115* |  | Putative response regulator (trancsriptional regulation) | 3.3 |
| *smb21079* |  | transcriptional regulator | -6.3 |
| *smb20668* |  | putative 3-hydroxyisobutyrate dehydrogenase, EC:1.1.1.31 (Val, Leu, and Ile metabolism) | -3.9 |
| *smb20622* |  | ATP-binding protein (putative simple sugar ABC transport system) | -5 |
| *smb20467* |  | Putative sensor kinase (trancsriptional regulation) | 4 |
| *smb20414* |  | hypothetical protein | 5.8 |
| *smb20231* |  | substrate-binding protein (putative multiple sugar transport system ) | -9.2 |
| *smb20227* | *ndiA-1* | nutrient deprivation-induced protein | 4.1 |
| *smb20007* | *katC* | catalase C protein, EC:1.11.1.6 (decomposition of hydrogen peroxide to water and oxygen) | 3.4 |
| *sma1711* |  | arginase, EC:3.5.3.1 (Arg and Pro metabolism) | 3.9 |
| *sma1427* |  | solute-binding protein ( putative simple sugar ABC transport) | 3 |
| *sma1386* |  | Oxidoreductase | -4 |
| *sma1096* |  | hypothetical protein | -4.2 |
| *sma1093* |  | hypothetical protein | 5.9 |
| *sma0747* |  | hypothetical protein | 6 |
| *sma0714* |  | ATP-binding protein (putative multiple sugar ABC transport) | 3.9 |
| *sma0134* |  | hypothetical protein | 3.6 |

Genes are grouped by replicon and ordered by descending gene number. FC (*dme/*wt) is the fold change of gene expression (*S. meliloti* glucose-grown cells of the *dme* mutant vs wild type). Significantly regulated genes: FC ≥ 3 and *P*-value (Student’s *t* test) ≤ 0.05. Minus (-) genes are significantly downregulated. wt – *S. meliloti* wild type strains RmP110; *dme* – *S. meliloti dme* mutant RmP2189.

**Table S5.** Differentially expressed genes in succinate-grown cells of a *S. meliloti tme* mutant compared with the wild-type strain.

| Gene ID | Gene name | Production description (functions) | FC (*tme/*wt) |
| --- | --- | --- | --- |
| *smc03858* | *pheAa* | chorismate mutase, EC:5.4.99.5 ( biosynthesis of Phe, Tyr, and Trp) | 3 |
| *smc03131* |  | periplasmic solute-binding protein (polar amino acid ABC transport system) | 12.7 |
| *smc03025* | *fliI* | Flagellum-specific ATP synthase, EC:3.6.3.14 (Cell motility) | -10.5 |
| *smc02874* |  | N-acetylmuramic acid 6-phosphate etherase, EC:4.2.1.126 (Amino sugar and nucleotide sugar metabolism) | 5 |
| *smc02586* | *helO* | ATP-dependent helicase (DNA replication, transcription, translation, and repair) | -3.5 |
| *smc02515* |  | hypothetical protein | 7.2 |
| *smc02448* | *mutT* | mutator protein 7,8-dihydro-8-oxoguanine-triphosphatase (DNA /RNA repair during replication and transcription) | -6.1 |
| *smc02328* |  | hypothetical protein | 3 |
| *smc02229* |  | acyl-CoA dehydrogenase, EC:1.3.99.- ( fatty acid metabolism) | 3.1 |
| *smc02224* | *chaA* | calcium/proton antiporter transmembrane protein | 3.2 |
| *smc02047* | *gcvT* | glycine cleavage system aminomethyltransferase T, EC:2.1.2.10 (metabolism of Gly, Ser, and Thr ) | 3.8 |
| *smc01992* |  | D-xylulose reductase, EC:1.1.1.9 (pentose and glucuronate interconversions) | 4.6 |
| *smc01954* |  | transcriptional regulator | 3.4 |
| *smc01658* |  | Ferrioxamine B specific ferric iron reductase (iron uptake) | 3.3 |
| *smc01508* |  | hypothetical protein | 8.4 |
| *smc01424* | *nthA* | nitrile hydratase subunit alpha protein, EC:4.2.1.84 84 (metabolism of Trp and Xenobiotics, Styrene, Aminobenzoate, and Fluorobenzoate ) | 3.6 |
| *smc01317* | *rpoB* | DNA-directed RNA polymerase beta chain, (transcription) | 3.4 |
| *smc01274* | *crcB* | signal peptide protein | 3.1 |
| *smc01095* | *mexF1* | multidrug-efflux system transmembrane protein (efflux pumps) | 3.4 |
| *smc01093* |  | Putative esterase/lipase ( fatty acid metabolism) | 3.4 |
| *smc01036* |  | hypothetical protein | 3.4 |
| *smc01009* |  | hypothetical protein | -10.2 |
| *smc00569* |  | hypothetical protein | 26.8 |
| *smc00559* |  | hypothetical protein | 5.7 |
| *smc00478* |  | hypothetical protein | 3.6 |
| *smc00397* |  | hypothetical protein | -5 |
| *smc00253* |  | signal peptide protein | 3.4 |
| *smc00045* | *cycF* | Probable Cytochrome c556 | 3.5 |
| *smb21550* |  | hypothetical protein | 3.5 |
| *smb21475* |  | hypothetical protein | 4.4 |
| *smb21182* |  | hypothetical protein | 6.4 |
| *smb21027* |  | hypothetical protein | -3.1 |
| *smb20876* |  | hypothetical protein | -6.1 |
| *smb20708* |  | methylated-DNA--protein-cysteine methyltransferase, EC:2.1.1.63 (repair of alkylated DNA) | 6 |
| *smb20494* |  | transcriptional regulator | -3.7 |
| *smb20375* |  | transcriptional regulator | -4.2 |
| *smb20373* |  | permease protein (putative TRAP-type dicarboxylate system) | 6.1 |
| *smb20342* |  | isoquinoline 1-oxidoreductase, EC:1.3.99.16 | 3.1 |
| *smb20275* |  | hypothetical protein | 6.2 |
| *smb20257* | *cyaM* | adenylate cyclase (conversion of ATP to cAMP) | -9.7 |
| *smb20243* |  | glycosyltransferase ( biosynthesis polysaccharides) | 3.8 |
| *Continued on following page* | | | |
|  | | | |
| *Supplemental Table S5 -Continued* | | | |
| Gene ID | Gene name | Production description (functions) | FC (*tme/*wt) |
| *smb20206* | *pqqC* | PQQ biosynthesis protein (methonal metabolism) | 3 |
| *smb20175* |  | hypothetical protein | 6.6 |
| *smb20174* |  | Putative cytochrome c | 4.4 |
| *smb20173* |  | methanol dehydrogenase large subunit (Methonal metabolism) | 9.8 |
| *smb20172* |  | cytochrome c protein | 3.1 |
| *smb20171* |  | Putative S-formylglutathione hydrolase, EC:3.1.2.12 (Methonal metabolism) | 3.8 |
| *smb20166* |  | hypothetical protein | 3.1 |
| *smb20100* |  | Putative dehydrogenase | 3.2 |
| *smb20036* |  | solute-binding protein (Putative TRAP-type quinic acid transport system) | 5.2 |
| *sma5015* |  | hypothetical protein | -3.1 |
| *sma5000* |  | hypothetical protein | -12.3 |
| *sma2353* |  | xanthine dehydrogenase YagR molybdenum-binding subunit, EC:1.17.1.4 (Purine metabolism) | 3.6 |
| *sma2351* |  | xanthine dehydrogenase YagS FAD-binding subunit, EC:1.17.1.4 (Purine metabolism) | 4.8 |
| *sma2349* |  | xanthine dehydrogenase iron-sulfur-binding subunit (Purine metabolism) | 5.3 |
| *sma2269* |  | hypothetical protein | 8.8 |
| *sma1907* |  | hypothetical protein | 3.8 |
| *sma1882* |  | transcriptional activator | 5.1 |
| *sma1863* |  | permease protein (Peptides/nickel ABC transport system) | 3.2 |
| *sma1817* |  | hypothetical protein | 3.3 |
| *sma1643* |  | hypothetical protein | -6.9 |
| *sma1406* | *ttuD3* | hydroxypyruvate reductase, (Gly, Ser and Thr metabolism) | 21.2 |
| *sma1394* |  | hypothetical protein | 5.2 |
| *sma1101* |  | hypothetical protein | -5.3 |
| *sma0800* |  | permease protein (spermidine/putrescine ABC transport ) | 4.8 |
| *sma0631* |  | hypothetical protein | 3.4 |
| *sma0237* |  | Dehydrogenase | 3.3 |
| *sma0171* |  | hypothetical protein | 3.1 |
| *sma0070* |  | simple sugar permease ( ABC transport system) | 11.1 |

Genes are grouped by replicon and ordered by descending gene number. FC (*tme/*wt) is fold change of gene expression (*S. meliloti* succinate-grown cells of the *tme* mutant vs wild type). Significantly regulated genes: FC ≥ 3 and *P*-value (Student’s *t* test) ≤ 0.05. Minus (-) genes are significantly downregulated. wt – *S. meliloti* wild type strains RmP110; *tme* – *S. meliloti tme* mutant RmP2179.

**Table S6.** Differentially expressed genes in glucose-grown cells of a *S. meliloti tme* mutant compared with the wild-type strain.

| Gene ID | Gene  name | Production description (functions) | FC  *tme/*wt |
| --- | --- | --- | --- |
| *smc04385* |  | Putative aldehyde dehydrogenase transmembrane protein | 3.4 |
| *smc04220* |  | transcriptional regulator | 10.4 |
| *smc04028* | *gltB* | glutamate synthase, EC:1.4.1.13 (Ala, Asp and Glu metab.) | 3.8 |
| *smc03773* |  | hypothetical protein | 3.2 |
| *smc01509* |  | hypothetical protein | 5.7 |
| *smc01316* | *rpoC* | DNA-directed RNA pol subunit beta, (Transcription) | 3.6 |
| *smc01186* |  | hypothetical protein | 3.6 |
| *smc01159* |  | oxidoreductase | 3.1 |
| *smc00796* |  | hypothetical protein | 3.2 |
| *smc00682* | *hipO1* | hippurate hydrolase, EC:3.5.1.32 (Phe metabolism ) | 3.9 |
| *smc00364* | *rplT* | 50S ribosomal protein L20 | 3.1 |
| *smb21486* |  | Putative permease | 3.3 |
| *smb21441* |  | Inosine-5′-monophosphate dehydrogenase, (synthesis of GTP from IMP) | 3.2 |
| *smb21032* |  | hypothetical protein | 3.8 |
| *smb20473* |  | hypothetical protein | 3.4 |
| *smb20331* |  | hypothetical protein | 6.4 |
| *smb20263* |  | hydroxyproline-binding protein (polar amino acid ABC) | 3.4 |
| *smb20251* |  | hypothetical protein | 6.2 |
| *smb20227* | *ndiA-1* | nutrient deprivation-induced protein | 3.5 |
| *sma2115* | *gst13* | glutathione S-transferase, EC:2.5.1.18 | 3.1 |
| *sma1978* |  | hydrolase | 6 |
| *sma1427* |  | solute-binding protein ( sugar ABC transport system) | 5.3 |
| *sma1093* |  | hypothetical protein | 3.9 |
| *sma1053* |  | hypothetical protein | 3.1 |
| *sma0431* |  | hypothetical protein | 4.4 |
| *sma0044* |  | hypothetical protein | 3.2 |

Genes are grouped by replicon and ordered by descending gene number. FC (*tme/*wt) is fold change of gene expression (*S. meliloti* glucose-grown cells of the *tme* mutant vs wild type). Significantly regulated genes: FC ≥ 3 and *P*-value (Student’s *t* test) ≤ 0.05. Minus (-) genes are significantly downregulated. wt – *S. meliloti* wild type strains RmP110; *tme* – *S. meliloti tme* mutant RmP2179.

**
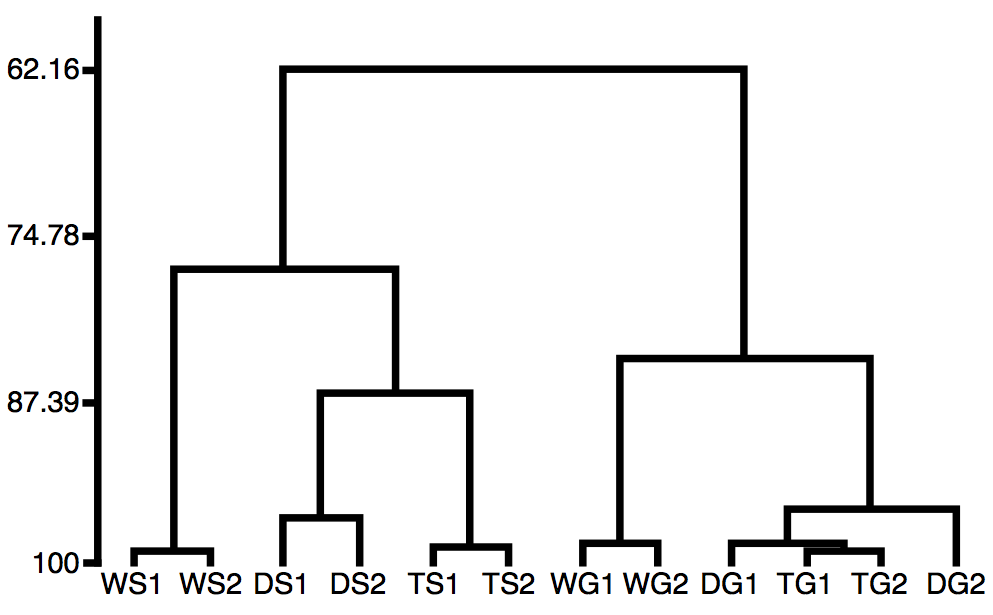
**

**Figure S1.** Hierarchical cluster analysis of the transcriptional profiles from *S. meliloti* grown in minimal MOPS-P2 medium with the carbon source succinate (S) or glucose (G). Strains are abbreviated as W for wild type RmP110, D for the *dme* mutant RmP2189 and T for the *tme* mutant RmP2179. The transcriptional profiles of the twelve *S. meliloti* cultures were clustered using Minitab 14 with Ward Linkage and Absolute Correlation Distance. Labels at the branch termini indicate the strain, carbon source, and replicate number; e.g. WS1 was wild type grown with succinate, replicate 1.
